# Assessing body composition via a smartphone computer vision application: High repeatability but method-dependent agreement compared with BODPOD and Inbody

**DOI:** 10.1371/journal.pdig.0000983

**Published:** 2026-09-02

**Authors:** Jahliya Sisohor, Jatin P. Ambegaonkar, Bryndan Lindsey, Qi Wei, Yosef Shaul, Joel Martin

**Affiliations:** 1 Sports Medicine Assessment Research & Testing (SMART) Laboratory, George Mason University, Virginia, United States of America; 2 State Key Laboratory of Mechanical System and Vibration, School of Mechanical Engineering, Shanghai Jiao Tong University, Shanghai, China; 3 Department of Bioengineering, George Mason University, Virginia, United States of America; 4 Center for the Advancement of Well-Being, George Mason University, Fairfax, Virginia, United States of America; National Tsing Hua University, TAIWAN

## Abstract

Accurate assessment of body composition is essential for monitoring health status, fitness progress, and disease risk. Traditional methods such as air displacement plethysmography (BODPOD) and bioelectrical impedance analysis (BIA) are widely used to estimate body fat percentage (BF%), fat mass (FM), and fat-free mass (FFM), but can be costly or inaccessible. Smartphone applications utilizing computer vision (CV) offer a promising alternative. This study evaluated the relative agreement and same session repeatability under standardized conditions of a smartphone-based CV application (CV_app_) compared to BODPOD and the InBody BIA device. Forty-nine adults (ages 18–70; 29 females, 27 racial and ethnic minority participants) completed two consecutive measurements using BODPOD, InBody, and CV_app_ in a single session. Differences in BF%, FM, and FFM estimates were analyzed using repeated-measures ANOVA. Agreement metrics included mean absolute error (MAE), root mean square error (RMSE), concordance correlation coefficients (CCC), and Bland-Altman analysis. Subgroup analyses examined differences by sex and minority status. The CV_app_ yielded higher BF% (mean=+2.2%, *P* = 0.004) and FM (+1.5 kg, *p* = 0.015) compared to BODPOD, and lower FFM than InBody (mean=−1.84 kg, *p* = 0.043). The CV_app_ agreement with BODPOD (MAE = 4.0, RMSE = 5.0, CCC = 0.86) was weaker than with the InBody (MAE = 3.3, RMSE = 4.3, CCC = 0.89), with wider limits of agreement. All methods showed excellent within session repeatability under standardized conditions (ICC > 0.99). A significant Sex×Method interaction was observed for BF% (*p* < 0.001), FM (*p* < 0.001), and FFM (*p* = 0.013), with females showing greater overestimation of BF% (mean=+3.9%) and FM (mean=+2.7 kg, *p* < 0.001), and underestimation of FFM (mean=−2.5 kg, *p* < 0.001) by the CV_app_. Among Minority participants, BF% and FM estimates from the CV_app_ were significantly higher than BODPOD (mean=+2.7%, *p* = 0.009; mean=+1.8 kg, *p* = 0.031), and FFM was lower (mean=−1.8 kg, *p* = 0.035), with significant Method×Minority Group interactions for FM and FFM (*p* < 0.03). These findings support cautious use of CV_app_-derived values for within-person monitoring under similar conditions but suggest they should not be treated as interchangeable with established comparator methods or used as standalone diagnostic estimates of body composition. Future efforts should address the need for more accurate, transparent, and equitable algorithms that are validated across diverse populations and testing contexts.

## Author summary

Body composition measurements are widely used to monitor health, fitness, and the effects of nutrition and exercise programs. Traditional assessment methods often require specialized equipment, trained personnel, and access to laboratories, making them impractical for many people. Smartphone applications that estimate body composition from photographs could make these assessments far more accessible, but they must first be shown to produce trustworthy results. In this study, we compared a commercially available smartphone computer vision application with two commonly used body composition methods under carefully standardized laboratory conditions. We found that the smartphone application produced highly consistent results when measurements were repeated during the same session, demonstrating excellent repeatability. However, its estimates were not interchangeable with the comparison methods and differed systematically for some groups of participants, particularly females and participants from racial and ethnic minority groups. These findings highlight an important distinction between consistency and agreement: a tool can provide repeatable measurements while still producing systematically different estimates. Our results suggest that smartphone-based body composition applications may be useful for monitoring changes within an individual over time, but they should be interpreted cautiously for estimating absolute body composition.

## Introduction

Body composition, which is the proportion of body fat (BF%), muscle, and other tissues in the body, is a key indicator of health [1] and physical performance [2,3]. Excess adiposity (*e.g*., obesity) is a well-recognized risk factor for chronic diseases such as type 2 diabetes, cardiovascular disease, and certain cancers [1]. The prevalence of obesity has risen dramatically and as of 2022, an estimated 16% of adults worldwide (~890 million people) were living with obesity [1]. In the United States, obesity now affects over 2 in 5 adults (41.9% in 2017–2020), reflecting a significant increase from previous decades [4]. Conversely, low BF% or low muscle mass can also carry health implications (*e.g.,* malnutrition or sarcopenia) [2], further emphasizing that body composition is central to health status. Beyond health and disease, body composition is crucial for performance in athletic [5,6] and occupational settings [3,7,8]. In clinical practice and research, having reliable methods to quantify body composition allows practitioners to better evaluate nutritional status [9], tailor interventions for weight management [10], or strength training [11], and predict functional outcomes [2,5].

A variety of methods are available to assess body composition, each with strengths and limitations [12], Historically, underwater weighing (*e.g.,* hydrostatic densitometry) was regarded as a gold-standard two-compartment model for body composition measurement [9,12]. However, due to practical limitations of hydrostatic weighing several methods have become more frequently used. One method is air displacement plethysmography (ADP), performed most commonly with the commercial BODPOD system. The BODPOD measures body volume by air displacement while a participant sits in a sealed chamber, and it computes body density and BF% using known equations (*e.g.,* Siri equation) [13]. The method is quick and non-invasive, and its accuracy in determining body composition is generally considered high [13]. For instance, ADP BF% estimates typically agree within a few percentage points of more direct methods [13]. Nonetheless, ADP has known sources of error [13]. Factors like clothing, air trapped in hair, or moisture on skin can affect the volume measurement and thus impact accuracy [13].

Another widely used body composition assessment method is bioelectrical impedance analysis (BIA) [14]. BIA estimates body composition by sending a low electrical current through the body and measuring the impedance, which varies between lean tissue (*i.e.,* high water content, more conductive) and adipose tissue (*i.e.,* low water, more resistive). Advantages of BIA include its portability, speed, and relatively low cost, making it one of the most popular methods in both clinical and fitness settings [15]. However, accuracy is a major concern with BIA [16]. The consistency of BIA results compared to reference techniques is variable as studies report that BIA can significantly diverge from ADP or dual-energy X-ray absorptiometry (DXA) measurements [14,17]. For example, BF% measured by BIA using the InBody 770 has been shown to be underestimated, on average, compared to values obtained from the BODPOD in young adults [16]. Moreover, BIA accuracy can be influenced by a person’s hydration status, recent food or exercise, and even the specific device algorithms used [14]. In addition to ADP and BIA, other techniques, such as DXA, Magnetic Resonance Imaging (MRI), or Computerized Tomography (CT) scans, can provide more detailed body composition data, including regional fat distribution and muscle mass, but these are costly, not portable, and typically limited to clinical or research facilities [12]. Thus, there is a need for methods that are both accurate and accessible.

Given the limitations of traditional techniques, recent efforts have turned to computer vision (CV) and digital anthropometry that leverage smartphone cameras to estimate body composition from digital images [18, 19]. The theoretical foundation of CV-based approaches lies in the relationship between external body shape and internal composition [20]. Modern CV algorithms can extract anthropometric information, such as body dimensions, contours, and volumes, which then serve as inputs to composition estimated. For example, CV algorithms can construct a 3D (3-Dimensional) avatar of a person from a series of smartphone digital photos, then calculate circumferences, volumes, and other shape metrics to estimate body composition metrics [21]. The benefits of CV-based body composition tools are substantial as they require minimal equipment – typically just a smartphone camera without specialized hardware. Such tools are also quick and non-invasive. For instance, a user can obtain results in minutes with only a few photos, avoiding the discomfort of the BODPOD enclosure or exposure to X-rays from DXA. Most CV approaches to estimate body composition use machine learning models to predict body fat or muscle metrics from image features [19]. For example, convolutional neural networks (CNNs) can be trained on thousands of labeled images to recognize subtle visual patterns of adiposity [22]. Other methods fit a statistical body shape model to the image, which can then be used to estimate tissue volumes [20,23].

More broadly, CV-based body composition estimation can be situated within the evolution of object detection and visual representation learning. Classical CV approaches relied on handcrafted features, such as edges, contours, shape descriptors, and texture patterns, to localize and characterize objects in images. Modern deep learning approaches, particularly CNN-based models, instead learn hierarchical visual representations directly from image data, enabling extraction of increasingly abstract spatial and semantic features. A recent review by Neha et al. [24] describes this transition from classical feature-based object detection to convolution-based models and highlights how learned visual representations have become central to modern image understanding. In the context of body composition estimation, these principles are relevant because smartphone-based CV tools must first represent body shape, boundaries, and image-derived anthropometric features before estimating BF%, FM, or FFM. Importantly, relying on body shape may present challenges to accurate predicting body composition of individauls with normal weight obesity [25], often referred to as ‘skinny fat’ [26]. Thus, although the proprietary CV_app_ evaluated in the present study does not disclose its internal architecture, its general task is conceptually aligned with broader CV pipelines that transform visual information into quantitative predictions. CV-based body composition estimation is also conceptually related to broader quantitative imaging paradigms such as radiomics. Radiomics refers to the extraction of high-dimensional quantitative features from medical images, including features related to shape, texture, intensity distributions, and spatial patterns, followed by feature selection, modeling, and validation. Although radiomics is most commonly applied to medical imaging modalities such as CT, MRI, and ultrasound, its central principle, transforming image-derived information into quantitative features for prediction, is closely aligned with CV-based digital anthropometry.

Several studies have evaluated the accuracy and reliability of smartphone or camera-based body composition estimation. Farina et al. [27] developed a smartphone computer vision application (CV_app_) that analyzes a single lateral full-body photograph to estimate fat mass (FM). In a sample of 117 adults, the application’s FM estimates showed a strong association with DXA (*R*^2^ = 0.95 for females, 0.91 for men) and no significant bias [27]. Affuso et al. [23] reported a method using two digital images per person combined with a support vector regression machine learning algorithm to predict BF%. In 226 adults, the photo-based model achieved a correlation of r = 0.87 with DXA BF%, with the mean BF% being within 0.1% indicating excellent agreement [23]. Tian et al. [20] introduced an algorithm to predict 3D body shape and composition from a single 2D photograph. The method used a frontal image to extract a silhouette, fit a 3D statistical shape model, and then estimated body composition from the 3D avatar. When evaluated against DXA in a validation set, the system was similar to photo-predicted and DXA-measured values of total body fat, and achieved high correlations (*R*^2^ = 0.81 in men, 0.74 in female for BF%). Importantly, Graybeal and colleagues found that for 3D scanning devices, the validity varied between sexes and racial and ethnic groups [18]. These findings provides evidence that demographic factors could influence body composition measurement accuracy when using digital imaging approaches.

More recently, studies have directly compared smartphone-based body composition tools to field methods in addition to gold-standard instruments [19,28,29]. For example, Majmudar et al. [29] validated a smartphone CV_app_ using CNNs in 134 adults, comparing it to DXA and multiple BIA devices, including smart scales and the BODPOD. The app showed high concordance with DXA (CCC = 0.93–0.96), no significant bias, and the narrowest limits of agreement (±5% BF%), outperforming other methods that showed greater bias and variability. In addition to accuracy, the reliability of smartphone-based body composition measures has been examined. Metoyer et al. [28] conducted an agreement study to test a smartphone body composition CV_app_ under various real-world conditions, such as different smartphone camera resolutions, lighting levels, and background colors. Interestingly, there was an effect of background color on BF% estimates, with non-white backgrounds yielding higher BF% estimates as compared to the white background condition. However, the BF% estimates under all tested conditions were strongly correlated with those under the reference condition [28].

Collectively, the literature suggests that smartphone-based CV_app_ methods for body composition can be accurate and reliable [19,28–30]. However, most prior studies have validated these tools against DXA [20,27,29] with lesser data existing on how a CV_app_ compares with other common methods like BODPOD and BIA or on the test–retest reliability of a CV_app_. Furthermore, whether smartphone-based CV_app_ assessments are accurate and reliably consistent across sexes and among individuals from racial and ethnic minority groups, an important consideration for equitable adoption remains relatively underexplored, and has been reported in other common methods of assessing body composition [31]. Therefore, the purpose of the current study was to assess the relative agreement and same-session repeatability of a smartphone-based body composition CV_app_ in comparison to two commonly use body composition methods, ADP (*i.e.,* BODPOD) and BIA (*i.e.,* InBody), under standardized laboratory conditions. Specifically, this study aimed to: 1) compare BF%, FM, and FFM estimates from the CV_app_ with those from BODPOD and InBody, evaluating relative agreement and method-dependent differences; 2) determine the same session repeatability of the CV_app_, under standardized conditions; and 3) examine whether method agreement differed across sex, racial, and ethnic groups. The current study was designed to evaluate whether CV_app_-derived BF%, FM, and FFM estimates are comparable to methods commonly used in research, clinical, fitness, and applied settings, rather than to establish absolute accuracy against a criterion reference method such as DXA or a 4-compartment model. The findings can help inform the responsible and inclusive use of such technology in health, performance, and telemedicine settings.

## Materials and methods

### Study design

A repeated-measures, single-session design was used to compare body composition estimates across three methods: 1) ADP via the BODPOD, 2) BIA via the InBody, 3) and a smartphone CV_app_ (the Body Composition Scanner, Spren Technologies, Asheville, NC, USA). Because neither BODPOD nor InBody was treated as a criterion reference method, analyses were interpreted as tests of relative agreement and method-dependent differences rather than absolute accuracy or validity. Each participant completed two consecutive measurements per method under standardized laboratory conditions to assess agreement and test-retest reliability. All assessments were conducted in the Sports Medicine Assessment Research & Testing (SMART) Laboratory at George Mason University University (IRB #: 1665548). The same researcher administered all assessments to minimize interrater variability.

### Participants

Forty-nine healthy adults (29 females, 20 males) between the ages of 21 and 70 years (mean ± SD: 37.2 ± 14.6 years) completed the study procedures. Participants’ self-reported race and ethnicity were recorded using U.S. Census classifications. A majority of the participants were White (American Indian or Alaskan Native, *n* = 0; Biracial/other, *n* = 2; Asian, *n* = 7; Black or African American, *n* = 7; Hispanic or Latino, *n* = 8; Middle Eastern or North African, *n* = 2; Native Hawaiian or Pacific Islander, *n* = 0; White, *n* = 22). Because the number of participants within several individual racial and ethnic categories was too small to support stable subgroup-specific modeling, participants were dichotomized for inferential analyses as racial/ethnic minority participants (i.e., all participants self-reporting as non-White; n = 27) or racial/ethnic non-minority participants (i.e., White participants; n = 22) [32]. This grouping was used as a pragmatic analytic strategy and should be interpreted as a coarse social-category comparison rather than as a direct measure of phenotypic characteristics relevant to CV performance. Inclusion criteria included age ≥ 18 years, no current pregnancy, and no implanted pacemakers. Participants were instructed to avoid exercise, alcohol, and excessive caffeine for 24 hours and to fast for 4 hours before testing, except for water, which could be consumed up to 45 minutes prior. The study protocol was approved by the university’s Institutional Review Board and all participants provided written informed consent prior to participation in the study.

### Protocol

**Fig 1:**
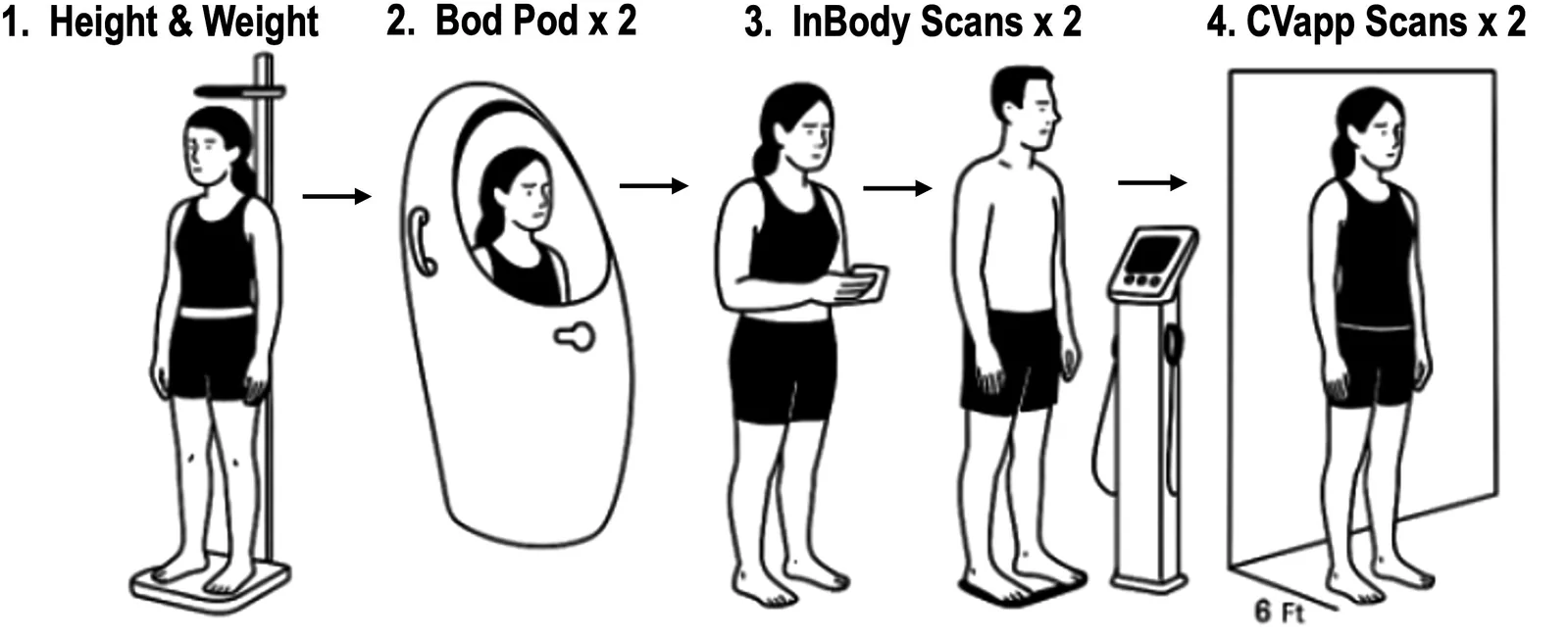
Study protocol (n = 49 participants). The body composition assessments were administered in a fixed sequence for all participants, with two consecutive measurements each for the BODPOD, InBody, and computer vision smartphone application. A total of 29 females and 20 males completed all procedures, including 27 participants who self-identified as members of racial and ethnic minority groups.

Upon arrival, participants changed into the required testing attire consisting of black spandex shorts and swim cap with sports bras for females. Each participant’s height and weight were measured using a stadiometer (Detecto, Webb City, MO, USA) and a digital scale (EatSmart, Tokyo, Japan) then these values were recorded and later entered into the devices or app as needed. The body composition assessments were administered in a fixed sequence for all participants with two consecutive measurements of: (1) BODPOD, (2) InBody, and (3) CV_app._ This order was selected to standardize participant flow, operator procedures, device setup, and imaging conditions within the single-session protocol (see Fig 1) and to maximize consistency while minimizing variation in test administration across participants. Because all three assessments were passive and completed consecutively, meaningful fatigue-related effects were considered unlikely. However, because the order was not counterbalanced, the potential influence of order effects cannot be fully excluded. For each method, the two measurements were completed consecutively with recalibration of the instrument between trials as appropriate. All CV_app_ measurements were performed in the same laboratory space with a plain white backdrop for photography and consistent ambient lighting. Throughout the session, the researcher followed identical procedures for every participant to minimize any variability in test administration and the same researcher tested all participants.

### Body composition measurements

#### Air Displacement Plethysmography (ADP).

Body composition was measured using a BODPOD GS-X system (COSMED USA Inc., Concord, CA, USA). The device was calibrated at the start of each day of testing. During each BODPOD trial, the participant sat inside the chamber wearing minimal attire and a swim cap. The system measured body volume twice and averaged the values, with thoracic gas volume predicted by the software. Body density was calculated from body mass and volume, and BF% was then derived using the Siri equation based on the measured body density. Each participant underwent two sequential BODPOD assessment measurements as described above.

#### Bioelectrical Impedance Analysis (BIA).

Body composition was also assessed using the InBody 570 device (InBody USA, Cerritos, CA, USA), which employs a tetrapolar 8-point tactile electrode system and multifrequency impedance analysis. Participants stood barefoot on the scale platform and gently held the hand electrodes. Prior to measurement, participants wiped hands and feet with the manufacturer’s electrolytic tissues to improve conduction. The device automatically measured the participant’s weight, while height from the stadiometer measurement was manually input by the researcher. The InBody then analyzed impedance to estimate BF%, FM, and FFM. Two sequential InBody measurements were completed for each participant, with the participant stepping off the platform and then re-resuming the testing position for the second measurement.

#### Computer Vision Smart Phone App.

The CV_app_ was used on an iPhone 13 Pro Max (Apple Inc., Cupertino, CA, USA) to estimate body composition from digital images captured with a 12-megapixel (MP) camera. The CV_app_ evaluated in this study was a commercially available smartphone-based body composition application. Although the user-facing acquisition workflow is described below per developer instructions, the internal CV model architecture, training dataset composition, preprocessing steps, feature extraction strategy, and inference mechanisms are proprietary and were not publicly disclosed by the developer.

Specifically, a profile was created for each participant in the app, and demographic data (age, sex, height, weight, and ethnicity) were entered before scanning. The smartphone was mounted on a tripod at a marked distance of 1.83 m (6 feet) and a height of 0.61 m (2 feet) from the participant. For each scan, two digital images of the front and right side views were captured with the participant standing upright against a plain white background under natural light. The standardized background and lighting was used to intentionally reduce environmental sources of image variability Table 1. The camera distance, background, and lighting were standardized for all app measurements, as consistent conditions are known to improve the accuracy of image-based body composition analysis (e.g., a white background yields higher agreement than a darker background) [28]. The CV_app_’s computer vision algorithm processed the images and generated estimates of BF%, FM, and FFM. Each participant completed two sequential measurements with the CV_app_.

**Table 1:** Summary of body composition measurement methods and capture conditions.

Method	Device (Manufacturer)	Participant Positioning	Key Capture/Testing Conditions	Primary Outputs
Air displacement plethysmography	BODPOD GS-X (COSMED USA Inc., Concord, CA, USA	Participant seated quietly inside the chamber wearing minimal clothing and a swim cap	Device calibrated at the start of each testing day; body volume measured twice within each trial; thoracic gas volume predicted by software; two sequential assessments completed	BF%, FM, FFM
Bioelectrical impedance analysis	InBody 570 (InBody USA, Cerritos, CA, USA)	Participant stood barefoot on the scale platform while holding hand electrodes	Hands and feet wiped with manufacturer-provided electrolytic tissues; height manually entered; device-measured body mass; participant stepped off and resumed testing position before the second assessment	BF%, FM, FFM
Smartphone computer vision application	Body Composition Scanner CVapp on iPhone 13 Pro Max (Spren Technologies, Asheville, NC, USA; Apple Inc., Cupertino, CA, USA)	Participant stood upright in standardized attire against a plain white background	12-megapixel smartphone camera; phone mounted on tripod at 1.83 m distance and 0.61 m height; front and right-side images captured; fixed smartphone model; standardized background and ambient lighting; demographic inputs entered into app; two sequential assessments completed	BF%, FM, FFM

**Abbreviations:** BF%, body fat percentage; CVapp, computer vision smartphone application; FM, fat mass; FFM, fat-free mass.

### Statistical analysis

Preliminary analyses involved data cleaning and descriptive statistics. The raw values were first screened for missing or implausible entries. Normality was assessed with Shapiro-Wilk tests and visual inspection of data distributions. For each method, test-retest data were averaged for descriptive and agreement analyses.

Our main analyses focused on assessing agreement between methods, test-retest reliability, and subgroup performance by sex and racial and ethnicity. The primary outcomes were BF%, FM, and FFM, and the primary method contrast was CV_app_ versus BODPOD, with CVapp versus InBody and BODPOD versus InBody considered secondary method contrasts. Agreement interpretation prioritized Bland–Altman mean differences and limits of agreement, along with Lin’s CCC, whereas Pearson correlations, MAE, RMSE, and ICC values were considered complementary agreement metrics. For reliability analyses, ICC was interpreted alongside absolute error metrics, including SEM, CoV, and CR, because relative consistency alone does not indicate accuracy or robustness across testing conditions. As described above participants were categorized dichotomously into Minority and Non-Minorities based on self-reported race and ethnicity. These subgroup analyses were intended to evaluate whether agreement differed across the analytic strata available in the present sample. Accordingly, race and ethnicity should not be interpreted as causal mechanisms, as no direct measures of skin tone, image contrast, body morphology, or regional fat distribution were available. Descriptive statistics were calculated for participant characteristics and for each body composition outcome by method and demographic variables.

To determine if the three methods yielded significantly different body composition measures, a one-way repeated-measures ANOVA on each outcome (BF%, FM, FFM), with method (3 levels: BODPOD, InBODY, CV_app_) as the within-subjects factor was conducted. Mauchly’s test was used to verify the sphericity assumption for each ANOVA; if violated, a Greenhouse-Geisser correction was used. When a significant main effect of method was found, pairwise post-hoc comparisons between methods were performed with Bonferroni adjustments. Bonferroni adjustments were applied within each outcome-specific family of pairwise method comparisons. Effect sizes for main effects were reported as generalized eta-squared (*η*²_G_) values and interpreted as 0.01 for *small*, 0.06 for *medium*, and 0.14 or greater for *large* effects [33]. Pair-wise effect sizes were reported as Cohen’s d and interpreted as small (*d* = 0.2), medium (*d* = 0.5), and large (*d* = 0.8) [33].

Agreement between methods was evaluated via Bland–Altman analysis: for each pair of methods, we calculated the mean difference in BF%, FM, and FFM between the two methods as well as the 95% LOA (computed as mean difference ± 1.96 SD of the differences) to assess the extent of agreement and to check for any systematic bias or proportional differences between methods. In addition, method agreement was further examined using Pearson correlation coefficients (*r*) to assess linear associations, mean absolute error (MAE) and root mean square error (RMSE) to evaluate average and squared deviations, Lin’s CCC to assess accuracy and precision [34], and intraclass correlation coefficients (ICC) using a two-way model for absolute agreement. CCC values were interpreted as <0.90 = *poor*, 0.90–0.95 = *moderate*, 0.95–0.99 = *substantial*, and >0.99 = *almost perfect* agreement [35]. ICC values were interpreted using the following thresholds: < 0.50 = *poor*, 0.50–0.74 = *moderate*, 0.75–0.89 = *good*, and ≥0.90 = *excellent* reliability [36].

Test–retest reliability for each measurement technique was assessed using multiple metrics. For each device/app, the two successive measurements of BF%, FM, and FFM were compared. We computed the ICC (2,1) for each method using a two-way random-effects model (single measures, absolute agreement) to quantify relative consistency between measurement 1 and 2. The standard error of measurement (SEM) and the coefficient of variation (CoV) for each method’s repeated measurements was computed to gauge absolute measurement error and within-method variability. SEM was calculated using the standard deviation (SD) of the differences between two repeated measurements, divided by the square root of two. This approach assumes equal error variance across measurements and provides an estimate of the typical measurement error inherent to the testing procedure. The CoV was calculated to express the SEM as a percentage of the overall mean of both measurements: CoV = (SEM / overall mean) x 100%. The ICC (2,1) was used to evaluate consistency between measurements. The Coefficient of Reliability (CR) was calculated as 1.96 times the SD of the differences, representing the range within which 95% of the differences between measurements are expected to occur.

Secondary subgroup analyses were conducted to examine whether body composition estimates differed by sex (male vs. female) and/or self-reported racial and ethnic minority status (Minority vs. Non-Minority). A series of repeated-measures ANOVA models were used to test for main effects of method and subgroup, as well as Method × Sex and Method × Minority Group interactions. Greenhouse-Geisser corrections were applied when Mauchly’s test indicated violations of sphericity. Follow-up pairwise comparisons of estimated marginal means were conducted using Tukey’s adjustment. To evaluate potential differences in participant demographics, a chi-square test of independence was used to assess the association between sex and Minority Group status. Statistical significance was set at α < 0.05 for all tests. All statistical analyses were performed using R (version 4.3.1).

## Results

Descriptive statistics of the participants are provided in Table 2. The BODPOD assessment indicated that the average BF% among participants was 24.6% (SD = 9.6%), with values ranging from 3.8% to 47.4%. Males were taller (t(48) = -4.89, p < 0.001, d = 1.40) and had greater body mass than females (t(48) = -4.52, p < 0.001, d = 1.29). Females exhibited higher BF% (*t*(48) = 3.16, *p* = 0.003, *d* = 0.91) and lower FFM (*t*(48) = -7.94, *p* < 0.001, *d* = 2.28) compared to males. However, FM did not differ between sexes (*t*(48) = 0.77, *p* = 0.45, *d* = 0.22). Participants identifying as members of a Minority Group were younger compared to Non-Minority participants (*t*(48) = -3.22, *p* = 0.002, *d* = 0.92). Minority and Non-Minority participants had similar height (*t*(48) = 0.15, *p* = 0.88, *d* = 0.04), body mass (*t*(48) = 0.33, *p* = 0.74, *d* = 0.10), BF% (*t*(48) = -0.42, *p* = 0.68, *d* = 0.12), FM (*t*(48) = -0.26, *p* = 0.80, *d* = 0.07), and FFM (*t*(48) = 0.45, *p* = 0.66, *d* = 0.13).

**Table 2:** Participant characteristics.

	All (*n* = 49) Mean ± SD [Range Min, Range Max]	Males (*n* = 20)	Females (*n* = 29)	Racial and Ethnic Non-Minorities (*n* = 22)	Racial and Ethnic Minorities (*n* = 27)
Age (years)	37.2 ± 14.6 [21, 70]	41.9 ± 15.8 [22, 70]	33.9 ± 13.1 [21, 68]	43.8 ± 15.2 [22, 70]	31.8 ± 11.9 [22, 70]
Height (cm)	171.3 ± 8.5 [158.2, 191.2]	177.0 ± 7.7 [161.5, 191.2]	167.3 ± 6.7 [158.2, 180.5]	171.3 ± 7.8 [158.2, 188.3]	171.2 ± 9.2 [158.9, 191.2]
Mass (kg)	77.2 ± 15.4 [50.6, 123.0]	86.9 ± 13.4 [71.2, 123.0]	70.5 ± 13.1 [50.6, 99.5]	76.9 ± 11.2 [55.5, 99.5]	77.4 ± 18.3 [50.6, 123.0]
BF% (%)	24.6 ± 9.6 [3.8, 47.4]	19.9 ± 8.4 [3.8, 38.8]	27.8 ± 9.2 [10.8, 47.4]	25.1 ± 11.0 [8.4, 47.4]	24.2 ± 8.6 [3.8, 38.8]
FM (kg)	19.2 ± 9.8 [2.9, 47.6]	17.9 ± 10.3 [2.9, 47.6]	20.1 ± 9.5 [6.0, 47.0]	19.6 ± 10.3 [6.0, 47.0]	18.9 ± 9.5 [2.9, 47.6]
FFM (kg)	57.7 ± 12.3 [38.3, 85.9]	68.7 ± 7.8 [55.5, 85.9]	50.2 ± 8.5 [38.3, 73.0]	57.1 ± 10.3 [42.4, 77.3]	58.2 ± 13.9 [38.3, 85.9]

**Note:** Values are Mean ± standard deviation and minimum and maximum values below in brackets for air displacement plethsography (*e.g.,* BODPOD). **Abbreviations:** BF%, body fat percentage; FM, fat mass; FFM, fat-free mass; SD, standard deviation; Min, minimum; Max, maximum.

### Agreement between methods

A repeated measures ANOVA revealed significant main effects of measurement method on all body composition outcomes (See Table 3). Compared to the BODPOD, the CV_app_ values were higher for BF% (p = 0.005, d = 0.47) and FM (p = 0.021, d = 0.40), and significantly lower FFM (p = 0.042, d = 0.37). When compared to InBody, the CV_app_ also produced higher estimates of BF% and FM, though these differences were not statistically significant after Bonferroni corrections (BF%: p = 0.051; FM: p = 0.078). The FFM estimate from CV_app_ was lower than that of InBody, but this comparison did not reach statistical significance after adjustment (p = 0.052, d = −0.35). In contrast, BF%, FM, or FFM were similar between BODPOD and InBody. A summary of post hoc comparisons is provided in Table 3. Although the main effects of method were statistically significant, generalized eta-squared values were small (η²G = 0.004–0.010), indicating that the overall method effect accounted for a relatively small proportion of variance in the full sample. Therefore, interpretation of agreement between methods considered both statistical significance and the magnitude of pairwise differences, effect sizes, agreement metrics, and limits of agreement.

**Table 3:** Repeated measures ANOVA results and post hoc comparisons for body composition measures.

Outcome	*F(2,98*)	Mauchly’s Test *p*-value	Main Effect *p*-value	*η*²_G_	Post Hoc Comparisons	Adjusted *p*-value	Cohen’s *d*
BF% (%)	7.66	0.079	<0.001	0.010	BODPOD - CV_app_	0.005	-0.47
					InBody - CV_app_	0.051	-0.35
					InBody - BODPOD	0.446	0.21
FM (kg)	5.23	0.377	0.005	0.005	BODPOD - CV_app_	0.021	-0.40
					InBody - CV_app_	0.078	-0.33
					InBody - BODPOD	1.000	0.06
FFM (kg)	3.65	0.014	0.0221	0.004	BODPOD - CV_app_	0.042	0.37
					InBody - CV_app_	0.052	0.35
					InBody - BODPOD	1.000	-0.11

**Abbreviations:** CV_app_, computer vision smart phone application; BF%, body fat percentage; FM, fat mass; FFM, fat-free mass; *η*²_G_, generalized eta-squared. **Notes:** Effect sizes for main effects were reported as generalized eta-squared (*η*²_G_) values and interpreted as 0.01 for *small*, 0.06 for *medium*, and 0.14 or greater for *large* effects. Pair-wise effect sizes were reported as Cohen’s d and interpreted as *small* (*d* = 0.2), *medium* (*d* = 0.5), and *large* (*d* = 0.8).

**Fig 2:**
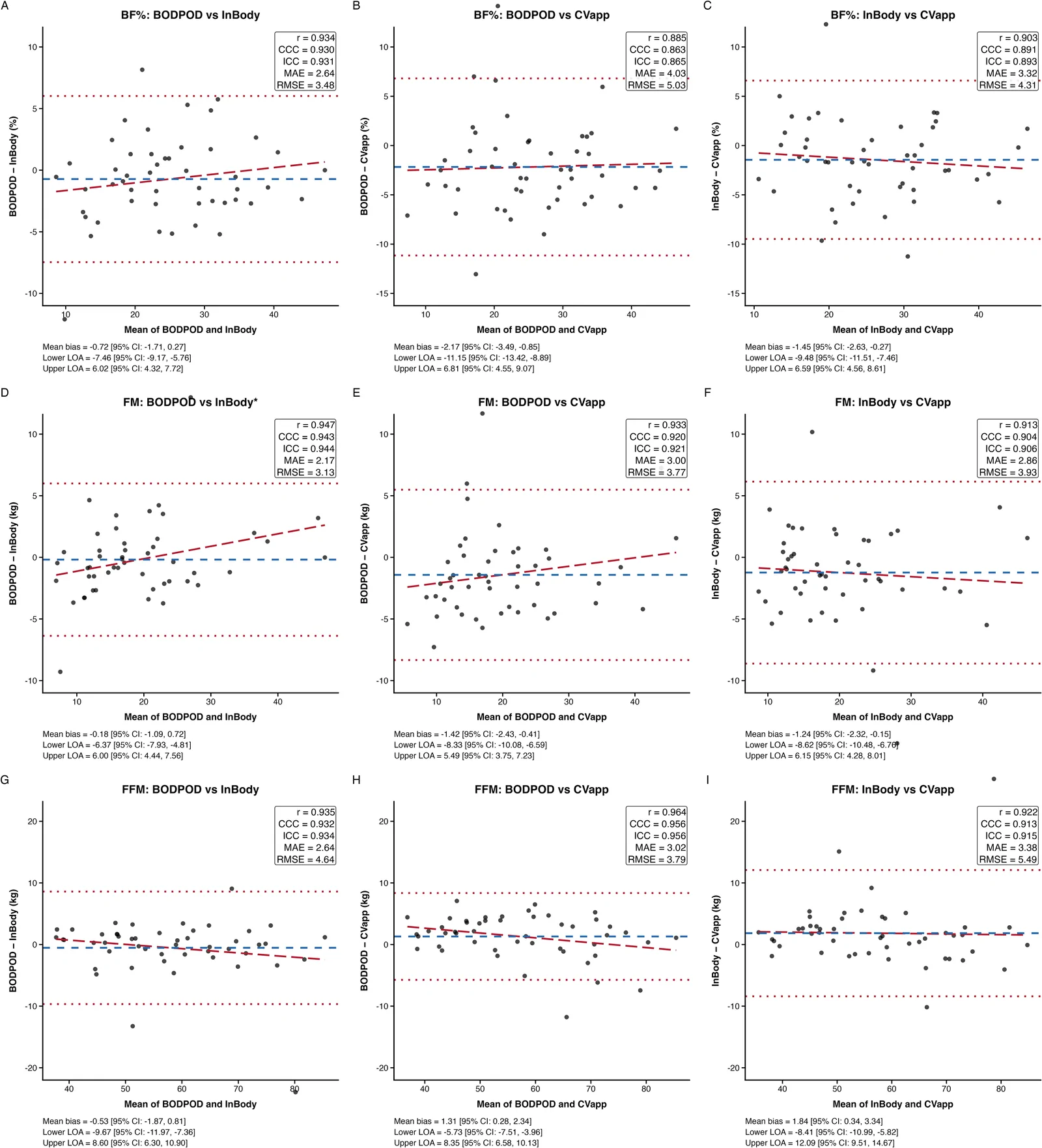
Bland Altman Plots Comparing Body Composition Methods. Dashed blue line denotes the mean bias. The dotted red lines denote the upper and lower limits of agreements. The dashed black line indicates the regression of the difference in methods versus the body composition metrics, which indicates the proportional bias. Asterisk at subplot title denotes statistically significant proportional bias (*p* < 0.05). Mean bias, limits of agreement (LOA), and 95% confidence intervals (CI) are presented in the bottom left of each plot. Additional agreement statistics include Pearson’s r (*r*), Concordance Correlation Coefficient (CCC), intraclass correlation coefficient (ICC), mean absolute error (MAE), and root mean square error (RMSE). Thresholds for interpreting agreement statistics are as follows: 1) CCC: values < 0.90 = *poor*, 0.90–0.95 = *moderate*, 0.95–0.99 = *substantial*, and > 0.99 = *almost perfect* agreement; 2) ICC (2,*k*): values < 0.50 = *Poor*, 0.50–0.74 = *Moderate*, 0.75–0.89 = *Good*, ≥ 0.90 = *Excellent* reliability. **Abbreviations:** CV_app_, computer vision smart phone application; CCC, Concordance Correlation Coefficient; ICC, Intraclass Correlation Coefficient; MAE, Mean Absolute Error; RMSE, Root Mean Squared Error; CV_app_, computer vision smart phone application; BF%, body fat percentage; FM, fat mass; FFM, fat-free mass.

Bland-Altman analyses further characterized agreement and method-dependent differences for BF%, FM, and FFM (Fig 2). Notably, wider LOA were observed when comparing the CV_app_ to BODPOD and InBody measures. For BF%, average CV_app_ values were higher than BODPOD- and InBody-derived values (BODPPOD: mean difference = -2.2%, lower LOA = -11.2%, upper LOA = 6.8%; InBody: mean difference = -1.4%, lower LOA = -9.5%, upper LOA = 6.6%) but with no evidence of proportional bias (p > 0.50 in both cases). For FM, the CV_app_ showed method-dependent differences with wide LOA compared to both BODPOD (mean difference = −1.5 kg, lower LOA = -8.3 kg, upper LOA = 5.5 kg) and the InBody (mean difference = −1.2 kg, lower LOA = -8.6 kg, upper LOA = 6.1 kg), reflecting slightly larger between-method variability. No proportional bias was detected for these comparisons (p > 0.20). For FFM, the CV_app_ values differed from BODPOD (mean difference = 1.3 kg, lower LOA = -5.7 kg, upper LOA = 8.4 kg) and InBody (mean difference = 1.8 kg, lower LOA = -8.4 kg, upper LOA = 12.0 kg). The BODPOD versus CV_app_ comparison indicated statistically significant proportional bias (p = 0.013), suggesting the magnitude of disagreement between methods varied across the range of FFM values.

Additionally, agreement metrics comparing body composition methods are presented on Fig 1. Agreement metrics indicated that the CV_app_ had lower concordance with BODPOD compared to InBody vs BODPOD across all body composition measures. While ICC values were generally high (≥0.86) across all comparisons the CCC indicated poor agreement between the CVapp with both the BODPOD and InBody. Additionally, error metrics (MAE, RMSE) were relatively similar when comparing CV_app_ to BODPOD versus InBody to BODPOD.

### Test-retest reliability

Test–retest reliability metrics for each method are presented in Table 4. All methods demonstrated excellent reliability across all outcome measures, with ICCs exceeding 0.99. These values should be interpreted as evidence of same-session repeatability under tightly controlled testing conditions, rather than evidence of accuracy or agreement with comparator methods. The CV_app_ exhibited the highest ICC values across the three methods, indicating near-perfect agreement between repeated measurements under standardized capture conditions and also demonstrated the lowest SEM and CoV. The InBody SEM and CoV values were slightly higher than those of the CV_app_ but consistently lower than the BODPOD for all three outcomes. The BODPOD, while still demonstrating excellent reliability, had comparatively higher variability to the other methods. Interestingly, the BODPOD had the highest CoV observed for BF% (2.7%) among all methods.

**Table 4:** Test-retest reliability of methods.

Method	Metric	ICC	SEM	CoV	CCC	CR
BODPOD	BF%	0.995	0.7%	2.7	0.995	0.02
	FM	0.998	0.5	2.5	0.997	1.39
	FFM	0.998	0.5	0.8	0.998	10.07
InBody	BF%	0.999	0.3%	1.1	0.999	0.01
	FM	0.999	0.3	1.1	0.999	0.70
	FFM	1.00	0.3	0.4	1.000	0.70
CV_app_	BF%	1.00	0.2%	0.6	1.000	0.00
	FM	1.00	0.1	0.6	1.000	0.37
	FFM	1.00	0.1	0.2	1.000	0.37

**Abbreviations:** CV_app_, computer vision smart phone application; BF%, body fat percentage; FM, fat mass; FFM, fat-free mass; ICC, Intraclass Correlation Coefficient; SEM, Standard Error of Measurement; CoV, Coefficient of Variation (%); CCC, Concordance Correlation Coefficient; CR, Coefficient of Reliability.

### Agreement between methods by sex

**Fig 3:**
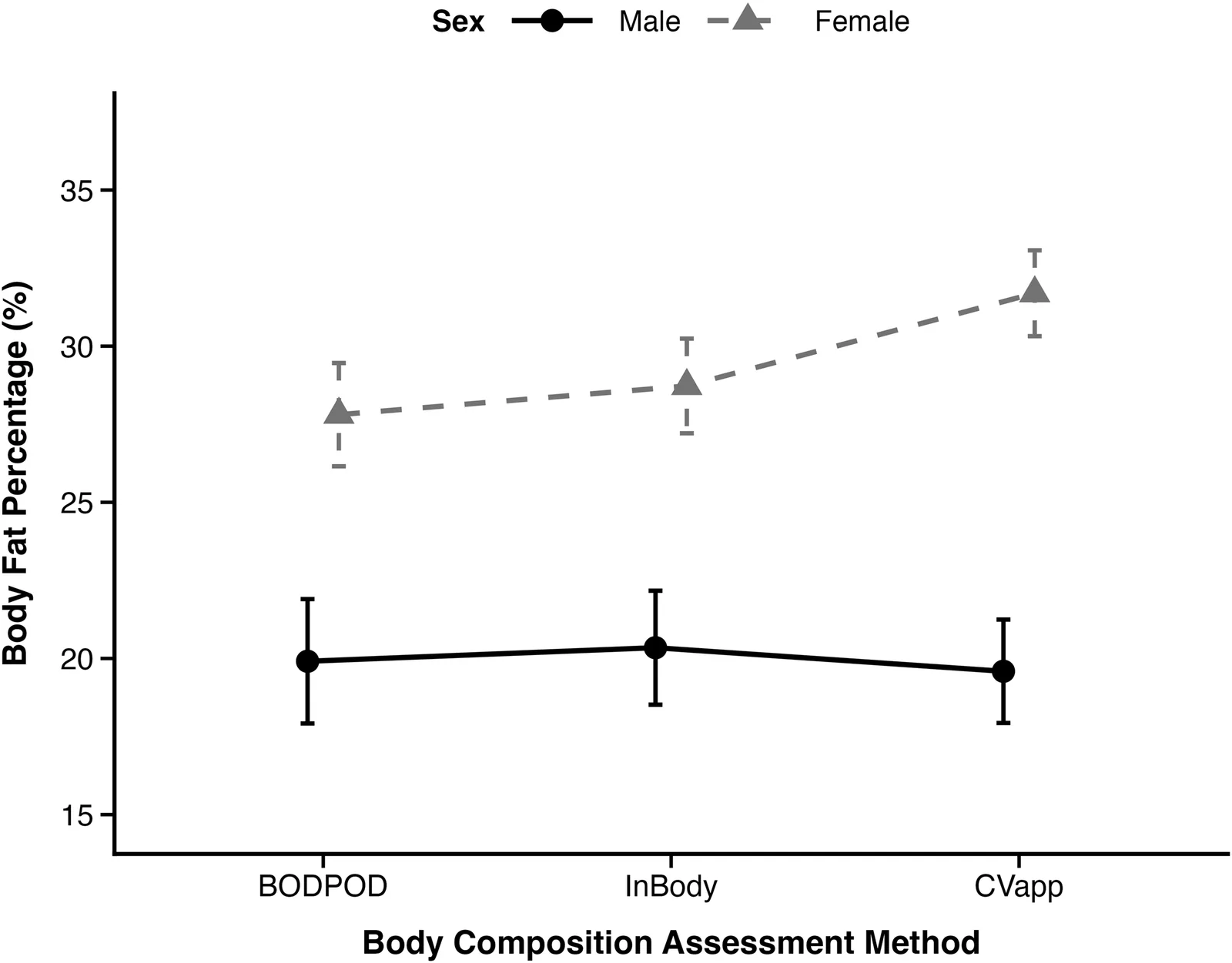
Interaction of Method and Sex on Body Fat Percentage. Abbreviations: CV_app_, computer vision smart phone application; M, Male; F, Female; BF%, body fat percentage.

**Fig 4:**
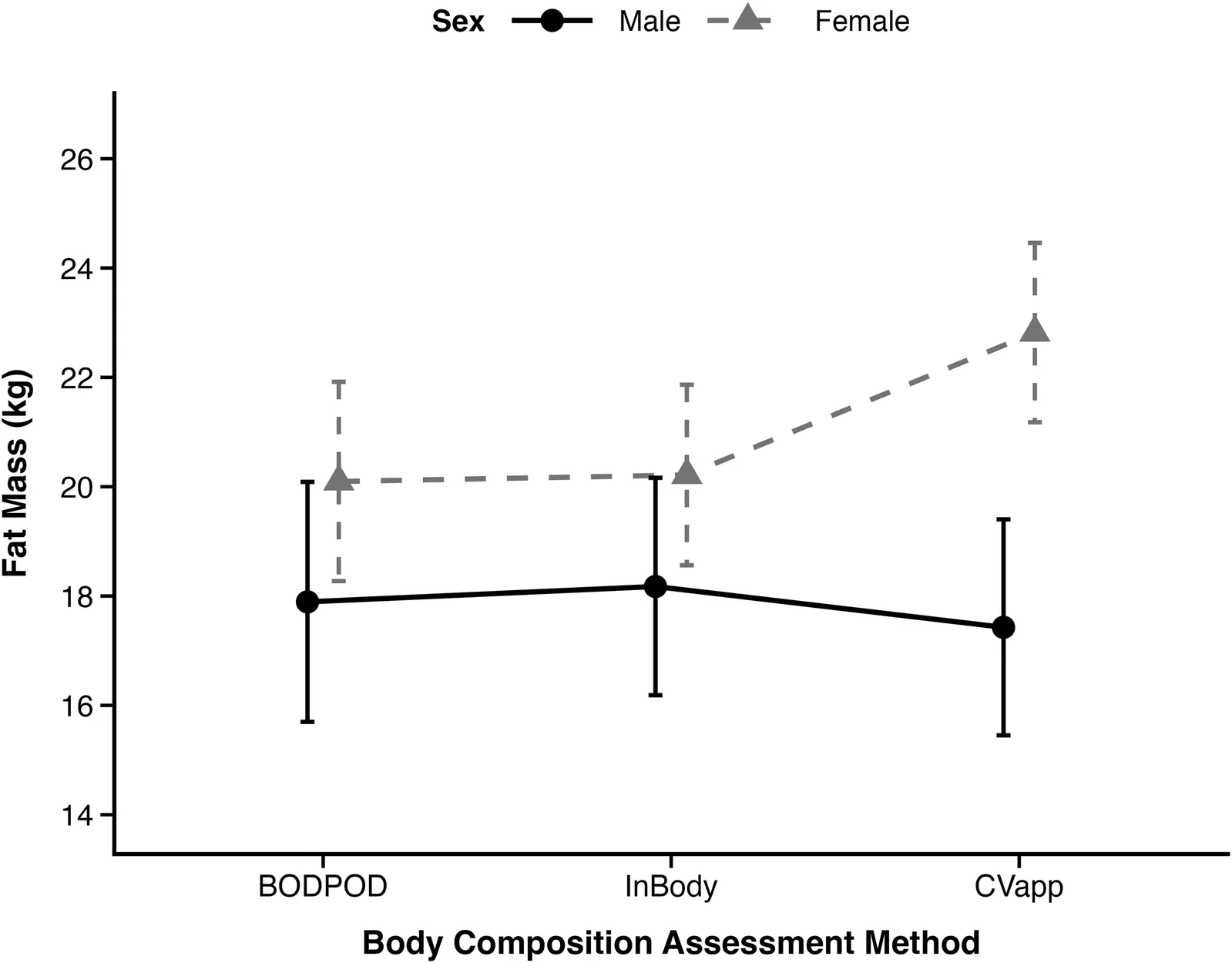
Interaction of Method and Sex on Fat Mass. Abbreviations: CV_app_, computer vision smart phone application; M, Male; F, Female; FM, fat mass.

**Fig 5:**
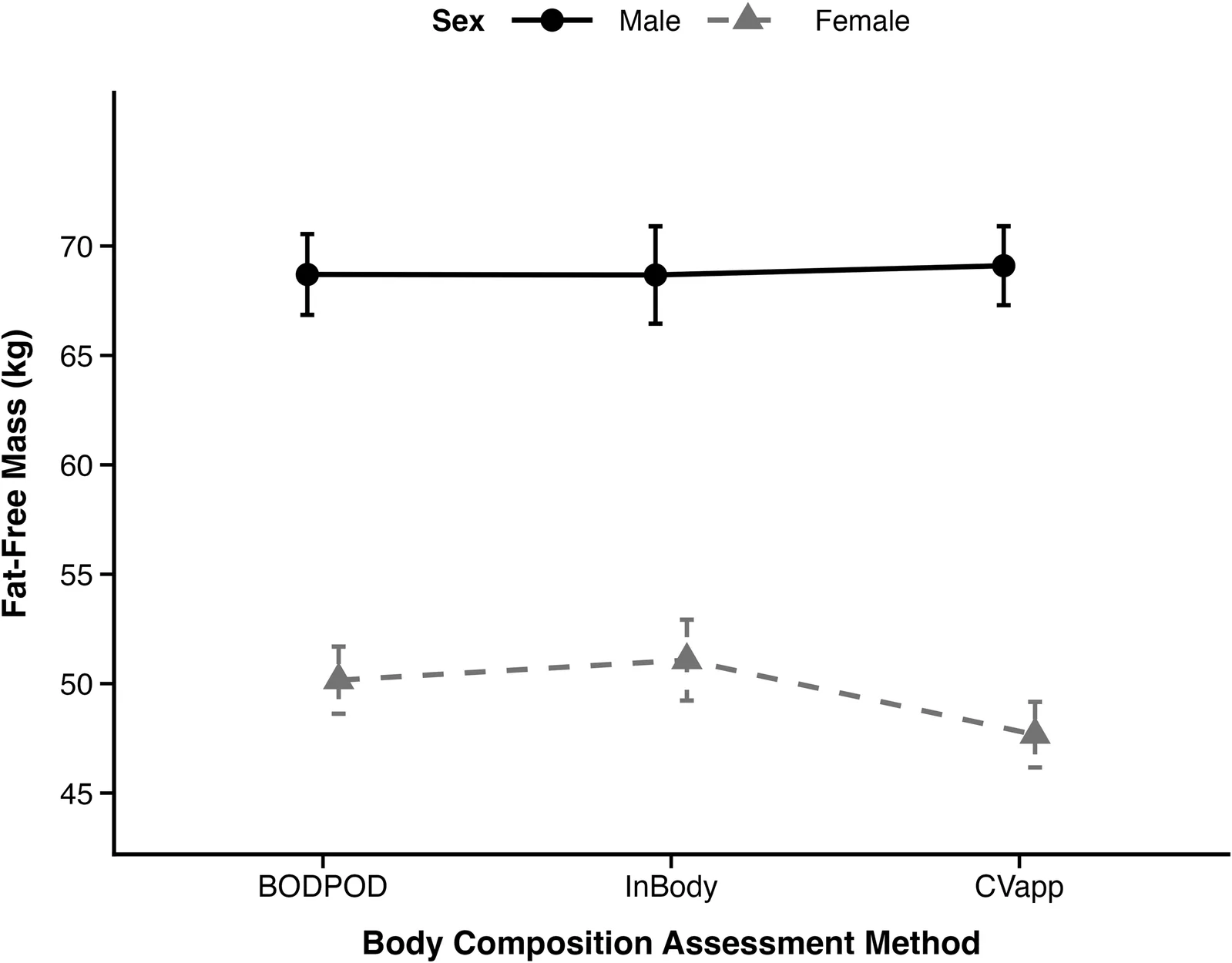
Interaction of Method and Sex on Fat-Free Mass. Abbreviations: CV_app_, computer vision smart phone application; M, Male; F, Female; FFM, fat-free mass.

The aggregate body composition data by sex and method are visually presented in Fig 3, Fig 4, and Fig 5, for body fat percentage, fat mass and fat-free mass, respectively.

#### Body fat percentage.

Significant main effects were found for Sex (*F*(1,47) = 17.04, *p* < 0.001, *η*²_G_ = 0.252) and Method (*F*(1.92, 90.22) = 5.40, *p* = 0.007, *η*²_G_ = 0.008) on BF%, as well as a significant Sex × Method interaction (*F*(1.92, 90.22) = 8.82, *p* < 0.001, *η*²_G_ = 0.013). Post-hoc comparisons showed that among females, the CV_app_ produced higher BF% values than both the BODPOD (mean difference = 3.9%, *p* < 0.001) and InBody (mean difference = 3.0%, p < 0.001), whereas there were no differences existed between BODPOD and InBody (mean difference = 0.9%, *p* = 0.340). In males, no differences in BF% were observed between methods (all *p* > 0.630).

#### Fat mass.

FM differed across method (*F*(1.98, 93.16) = 3.22, *p* = 0.045, *η*²_G_ = 0.003), but not across sex (*F*(1, 47) = 1.51, *p* = 0.226, *η*²_G_ = 0.030). Accordingly, the interaction between Sex × Method was significant (*F*(1.98, 93.16) = 7.94, *p* < 0.001, *η*²_G_ = 0.007), indicating that the difference in FM estimates across methods varied by sex. Post hoc comparisons within sex showed that, in females, the CV_app_ FM values were higher than both the BODPOD (mean difference = 2.72 kg, *p* < 0.001) and InBody (mean difference = 2.60 kg, *p* < 0.001), while BODPOD and InBody did not significantly differ. In males, no differences existed between any of the methods (*p* > 0.59). Comparisons between sexes within each method indicated a significant sex difference only for the CV_app_, with females having higher FM estimates than males (mean difference = 5.39 kg, *p* = 0.041).

#### Fat-free mass.

Males had greater FFM than females (*F*(1, 47) = 61.31, *p* < 0.001, *η*²_G_ = 0.545). The main effect of Method only approached significance (*F*(1.69, 79.62) = 2.90, *p* = 0.069, *η*²_G_ = 0.005). However, a significant interaction between Sex × Method was observed (*F*(1.69, 79.62) = 4.94, *p* = 0.013, *η*²_G_ = 0.009), indicating that the difference in FFM estimates between methods varied by sex. Post hoc comparisons within sex indicated that among females, the CV_app_ FFM values were lower than both the BODPOD (mean difference = −2.49 kg, *p* < 0.001) and InBody (mean difference = −3.41 kg, *p* = 0.002), while BODPOD and InBody did not differ. Among males, no differences were found between any of the methods (*p* > 0.85). Across all three methods, females had lower FFM than males, with sex differences ranging from −17.6 kg to −21.4 kg (all *p* < 0.001), consistent with physiological expectations.

### Agreement between methods by race and ethnicity analytic groups

**Fig 6:**
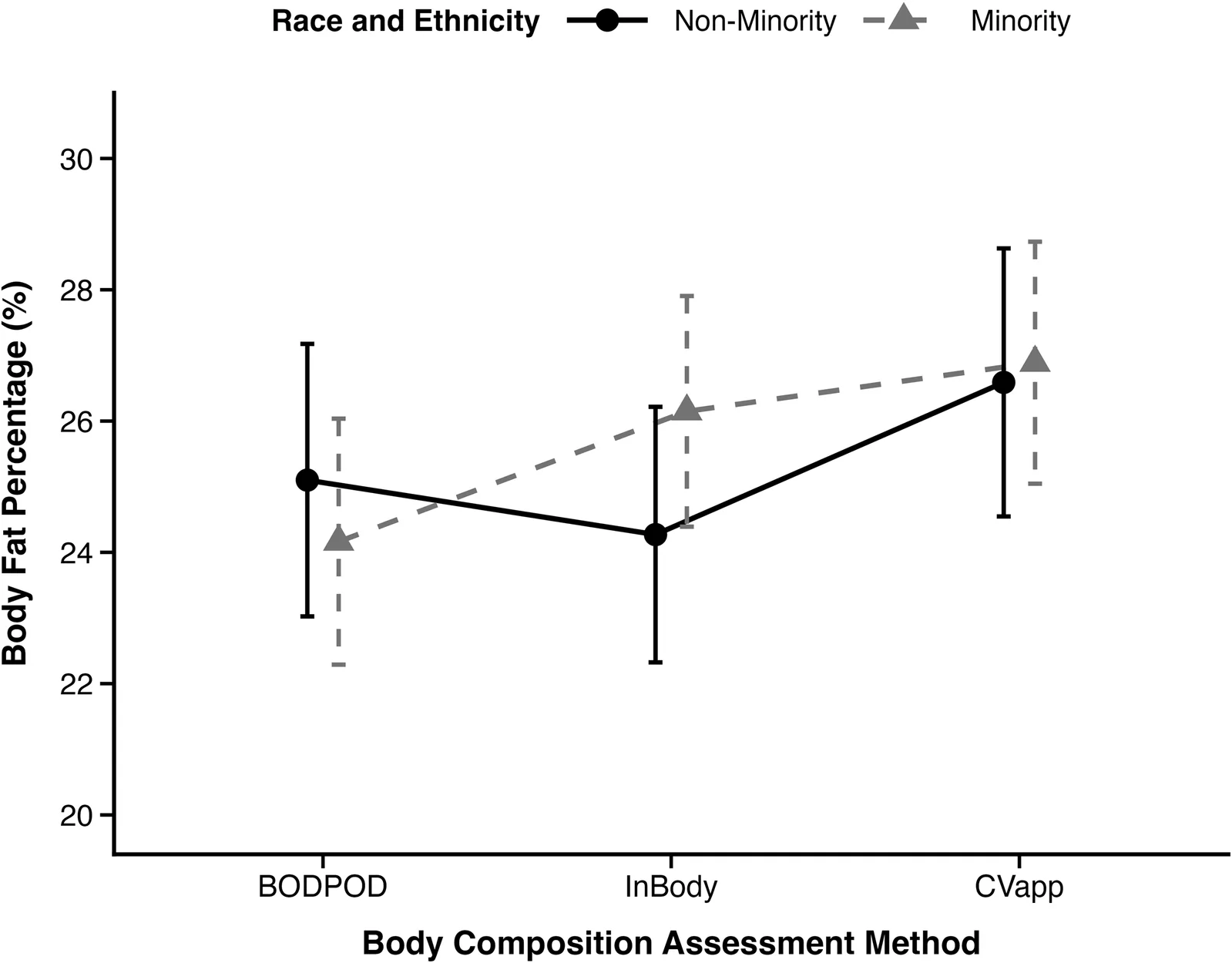
Interaction of method and racial and minority group on body fat percentage. Abbreviations: CV_app_, computer vision smart phone application; BF%, body fat percentage.

**Fig 7:**
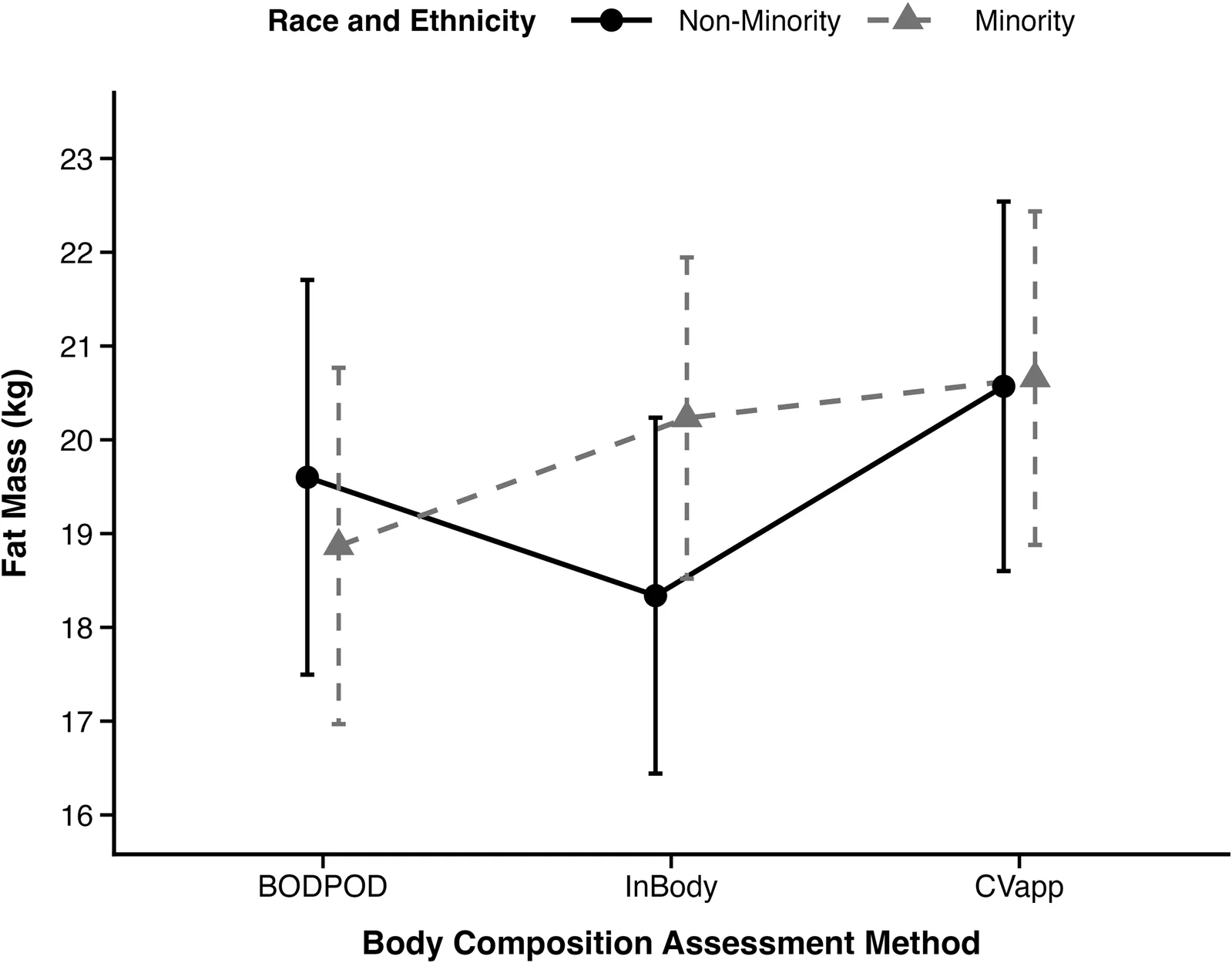
Interaction of method and racial and minority group on fat mass. Abbreviations: CV_app_, computer vision smart phone application; FM, fat mass.

**Fig 8:**
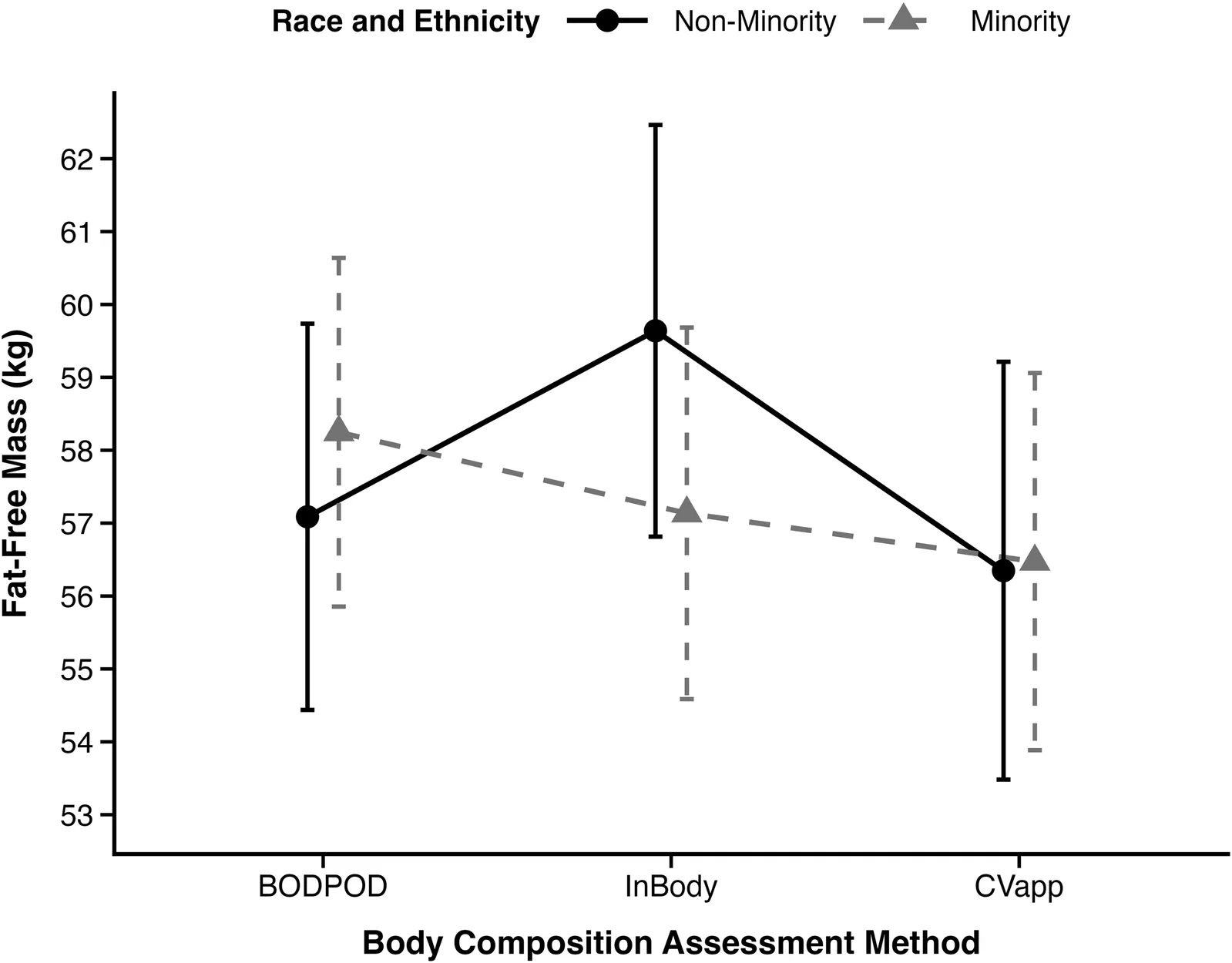
Interaction of method and racial and minority group on fat-free mass. Abbreviations: CV_app_, computer vision smart phone application; FFM, fat-free mass.

The aggregate body composition data by race and ethnicity and method are visually presented in Figs 6–8 for body fat percentage, fat mass and fat-free mass, respectively.

#### Body Fat Percentage.

BF% estimates differed across methods (F(2, 94) = 7.25, p = 0.001, *η*²_G_ = 0.009), but not across Minority Group (F(1, 47) = 0.02, p = 0.88, *η*²_G_ < 0.001), and although the Method × Minority Group interaction was not significant (F(2, 94) = 3.04, p = 0.053, *η*²_G_ = 0.004), there was a trend toward greater differences in measurement agreement for the Minority analytic group. Post hoc comparisons revealed that for the Minority participant group, BF% values from the CV_app_ were higher than BODPOD (+2.73%, p = 0.009), and BODPOD was lower than InBody (–1.99%, p = 0.006). Among Non-Minority participants, BF% from the CV_app_ was higher than InBody (+2.32%, p = 0.027). However, BF% did not differ significantly between groups within any individual method (all p = 0.47).

#### Fat Mass.

Average FM did not differ between groups (*F*(1, 47) = 0.03, *p* = 0.875, *η*²_G_ < 0.001), but did differ across Method (*F*(2, 94) = 5.15, *p* = 0.008, *η*²_G_ = 0.005). This observation was qualified by a significant Minority Group × Method interaction (*F*(2, 94) = 3.80, *p* = 0.026, *η*²_G_ = 0.004), suggesting that the differences among methods varied by group. Post-hoc pairwise comparisons revealed that, among Minority participants, FM estimates from the CV_app_ were higher than BODPOD (mean difference = 1.79 kg, *p* = 0.031), and BODPOD was lower than InBody (mean difference = −1.36 kg, *p* = 0.047). There was no significant difference between CV_app_ and InBody (*p* = 0.822). Among Non-Minority participants, FM from CV_app_ was higher than InBody (mean difference = 2.23 kg, *p* = 0.018). However, comparisons between CV_app_ and BODPOD (*p* = 0.410) and BODPOD and InBody (*p* = 0.113) were not statistically significant. There were no significant differences in FM between Minority and Non-Minority participants within any method (all *p* > 0.46).

#### Fat-Free Mass.

Overall FFM did not differ by group (*F*(1, 47) = 0.01, *p* = 0.9, *η*²_G_ < 0.001). A significant main effect of Method was observed (*F*(2, 94) = 5.04, *p* = 0.008, *η*²_G_ = 0.004), and a significant Minority Group × Method interaction emerged (*F*(2, 94) = 4.49, *p* = 0.014, *η*²_G_ = 0.004), suggesting the differences among methods varied across groups. Among Minority participants, FFM was lower when estimated by the CV_app_ compared to BODPOD (mean difference = −1.78 kg, *p* = 0.035). No other pairwise comparisons were significant (*p* > 0.38). Among Non-Minority participants, FFM estimates were lower using the CV_app_ compared to InBody (mean difference = −3.29 kg, *p* = 0.011), and BODPOD also reported lower FFM than InBody (mean difference = −2.55 kg, *p* = 0.022). No difference existed between CV_app_ and BODPOD t (*p* = 0.602). No significant differences existed in FFM between Minority and Non-Minority groups within any of the three methods (all *p* > 0.51).

To assess whether the distribution of sex differed across the minority analytic groups a chi-square test was conducted. There was no significant association between sex and minority group status, (χ²(1, N = 49) = 0.00, p = 1.00). Thus, the distribution of participants across sex (F, M) was similar for Minority (16 F, 11 M) and Non-Minority (13 F, 9 M) groups.

## Discussion

The primary purpose of this study was to evaluate the agreement and same-session repeatability of a smartphone-based CV_app_ for estimating body composition by comparing it with commonly used methods in clinical settings, and to assess whether average values and agreement differed by sex and racial/ethnic group. For the overall sample, CV_app_-derived BF% and FM values were higher, whereas FFM values were lower compared to thosefrom BODPOD and InBody. However, subgroup analyses revealed that these differences were influenced by sex and minority group status. Statistically significant method-dependent differences in body composition metrics were found for females but not males. The interaction between the Minority Group and Method indicated that agreement between the CV_app_ and comparator methods differed for Minority participants. Specifically, CV_app_-derived FM values were higher, and FFM values were lower than BODPOD-derived values among Minority participants, but not among Non-Minority participants. Interestingly, the interaction of Minority Group and Method did not reach statistical significance for BF%. However, these findings should be only be interpreted as differential method agreement across the analytic strata used in this study, rather than as evidence that race or ethnicity directly caused differences in CV_app_ performance. This is due to the using participant self-reported race and ethnicity, instead of using researcher-determined methods to classify parrticipants, such as skin color.

The CV_app_ had excellent test–retest reliability, indicating that the CV_app_ produces highly repeatable measurements although the CV_app_ showed lower agreement with comparator methods in certain subgroups. Importantly, these findings demonstrate that high repeatability does not necessarily imply strong agreement with comparator methods; the CV_app_ produced highly consistent repeated values, but those values differed systematically from BODPOD and InBody estimates in the overall and subgroup analyses. Because several statistically significant method effects were accompanied by small generalized eta-squared values, the practical importance of these findings should be interpreted in conjunction with the observed mean differences, pairwise effect sizes, CCC values, and limits of agreement rather than statistical significance alone.

Previous studies of smartphone computer-vision body composition tools have generally reported high accuracy and minimal bias [20,21,23,27,29]. Notably, Majmudar et al. [29] evaluated a 2D (2 Dimensional) digital image smartphone application versus DXA in 134 adults and found very low MAE in BF% (~2–3%) across the entire sample. Bland–Altman analysis showed computer vision had no significant bias and the narrowest limits of agreement (LOA; ± 5% fat) with DXA, in contrast to the other methods which exhibited systematic biases and wider variability [29]. Errors were only slightly higher in females (MAE = 2.3%) than men (MAE = 1.9%), and modestly higher in Black versus White participants (BF% MAE 2.7% vs 2.0%) [29]. In that study, the CV_app_ showed strong concordance with DXA for both sexes and no significant systematic error by demographic subgroups [29]. As previously described, Farina et al. [27] and Affuso et al. [23] also reported evidence supporting that 2D digital image approaches obtained with a smartphone camera can be used to estimate BF%, with strong correlation to DXA BF% values. Similarly, A 3D smartphone-scanning approach (non-rigid avatar reconstruction) also yielded extremely high reliability (ICC = 0.996–0.997) and showed no evidence of proportional bias in BF% estimates for two BF% estimation equations [30]. Tinsley and colleagues found that two estimation equations were statistically equivalent to DXA within a ± 2% margin and showed strong linear associations (Pearson’s r and CCC = ~0.90) [30]. The average MAE was approximately 3.5%, with a SEM around 4.2% across equations [30].

In contrast, Graybeal et al. [18] evaluated the validity and reliability of body composition estimates generated by smartphone applications against a 4-compartment criterion model in a diverse sample of adults (n = 184; 114F, 70M). In this study only one smartphone application (HALO) demonstrated equivalence for BF% with the 4-compartment model, while all smartphone applications showed proportional bias for BF% and varying degrees of error for FM and FFM [18]. Notably, the accuracy of body composition estimates from smartphone applications varied by sex and racial/ethnic group, likely due to differences in the underlying algorithms and the demographic composition of the data used to train them [18]. It should be noted that the smartphone CV_app_ used in the present study was not investigated in the aforementioned studies, which suggests that machine learning algorithm and training data could explain differences in the findings. Despite these limitations in validity, all smartphone application based methods demonstrated excellent reliability (ICCs ≥ 0.99) [18]. Together, the prior findings [18,29,30] when combined with the current study findings suggest that while smartphone CV-based smartphone applications can be highly reliable and accurate in ideal conditions to assess body composition, the results can vary depending on the application used and the user demographic characteristics.

### Practical implications

The current observations of method-dependent differences in have important practical implications. In consumer health or clinical settings, systematic method-dependent differences could lead to inappropriate advice or a loss of trust in the technology. The CV_app_’s high reliability means it could be useful for tracking relative changes over time (e.g., weight-loss progress) within an individual, provided that repeated measurements are collected under similar conditions and interpreted as within-person trends rather than absolute indicators of body composition accuracy. However, because CV_app_-derived values systematically differed from comparator-method values, practitioners should be cautious when using CV_app_ for baseline assessments or cross-sectional comparisons as a replacement for current body composition assessment methods. Specifically, higher CV_app_-derived BF% values in females and minority participants could lead to misclassification of health risk or unnecessary concern, while lower CV_app_-derived FFM values might obscure loss of lean mass. Furthermore, differences in subgroup-dependent methods could raise equity concerns if similar patterns are confirmed in larger and more granular validation studies. AI-driven tools that underperform for certain groups can exacerbate health disparities [37,38]. For example, facial-recognition studies have shown substantially smaller error rates for light-skinned males versus dark-skinned females [39,40]. Similar skin-tone related issues are also present with wrist-worn wearable devices [41]. In the context of the current study, a body-composition app that shows lower agreement with comparator methods across sex or race/ethnicity analytic groups may create a risk of unequal interpretation or use. Health technology developers and regulators should thus require subgroup validation to ensure objectivity before wide deployment [37,38]. Despite these issues, computer vision-based methods offer clear advantages (e.g., low cost, accessibility, no radiation, etc.) and could serve as screening or monitoring tools when more advanced methods (e.g., DXA, MRI) are unavailable [42]. For example, digital anthropometry has been advocated as a convenient pre-screening step [42]. Our results suggest, however, that any such screening should come with caveats, which clinicians and users must be aware that CV_app_-derived values may differ systematically across participant subgroups.

The magnitude of the observed method-dependent differences is also important when considering potential use cases. Body composition estimates are commonly used to inform nutritional assessment, health-risk stratification, sport and performance monitoring, and intervention decision-making [9,12]. For low-stakes screening or self-monitoring, the CV_app_ may be useful for identifying individuals who may benefit from follow-up assessment, provided results are interpreted as approximate estimates rather than diagnostic values. However, CV_app_-derived BF% values were 3.9% higher than BODPOD in females and 2.7% higher than BODPOD among racial and ethnic minority participants. Differences of this magnitude may be clinically relevant when individuals are near sex-, age-, or setting-specific classification thresholds, particularly because BF% classification ranges have been proposed for health-risk interpretation and may influence counseling or referral decisions [43,44]. Therefore, CV_app_-derived values should not be used as standalone diagnostic measures or as the sole basis for obesity classification, nutrition counseling, intervention evaluation, or occupational fitness decisions without confirmation from a validated criterion or comparator method.

### Limitations and Future Research Directions

This study has certain limitations that warrant consideration. A key limitation is that the study did not include a criterion reference method such as DXA or a 4-compartment model. While both the BODPOD and InBody are frequently used in research and applied settings, they have known errors and limitations [13,16,45]. For instance, the BODPOD can overestimate BF% in very lean individuals and underestimate BF% in obese subjects [46]. Thus, some of the CV_app_’s discrepancies might partly reflect reference-method limitations. Future work could compare the CV_app_ directly to DXA [29,30] or 4-compartment model [18] measurements to better distinguish comparator-method disagreement from CV_app_-specific error. A further limitation is that the CV_app_ is a proprietary commercial tool. The model architecture, training dataset distribution, body segmentation approach, feature extraction pipeline, and inference mechanisms were not publicly available. As a result, the present study cannot determine whether the observed subgroup-dependent method differences reflect training data imbalance, segmentation performance, model architecture, image preprocessing, or other deployment factors. Therefore, explanations related to training data representation, segmentation errors, skin tone, body morphology, or smartphone image processing should be interpreted as plausible mechanisms rather than empirically confirmed causes. Future studies should use transparent model-development designs or controlled validation experiments that systematically manipulate or account for lighting, background, camera characteristics, skin tone, body morphology, clothing, and subgroup-specific prediction error. Such designs would allow investigators to determine whether subgroup-dependent disagreement is driven primarily by image-capture conditions, segmentation performance, training data imbalance, model architecture, or post-processing/calibration procedures. This lack of transparency is a broader challenge in evaluating commercial AI-enabled health tools and reinforces the importance of independent validation across demographic and phenotypic groups. This is a similar challenge in radiomics research, which has highlighted persistent challenges in feature robustness, reproducibility, domain shift, validation bias, and clinical translation, which are also relevant to evaluating proprietary CV-based body composition tools across demographic subgroups [47].

We also note that our sample size could be considered limited [18,29,30], which may limit generalizability. The observed subgroup-dependent method differences may reflect several plausible mechanisms, including differences in training data representation, segmentation performance across skin tone or body shape, model assumptions about body composition distribution, or deployment-specific image preprocessing. These mechanisms could not be isolated in the present study and should be investigated in future work.

An additional limitation is that all assessments were completed in a fixed order: BODPOD, followed by InBody, followed by the CV_app_. This sequence was selected to standardize participant flow, operator procedures, and device setup within the single-session protocol. Because the assessments were passive and involved sitting quietly in the BODPOD, standing on the InBody platform, and standing for smartphone images, fatigue-related carryover was considered unlikely. However, the fixed sequence prevents method effects from being fully separated from potential order effects, and a counterbalanced design would have allowed these effects to be evaluated directly. Similarly, the CV_app_ was evaluated under standardized laboratory imaging conditions, including a fixed smartphone model, fixed tripod distance and height, plain white background, and consistent ambient lighting. These conditions were intentionally selected to minimize environmental sources of image variability and to evaluate relative agreement under optimized capture conditions. Therefore, the present findings should not be interpreted as representing CV_app_ performance across all possible home or field-use conditions. Rather, the standardized design provides a conservative evaluation of whether method-dependent differences are present even when image-capture variability is minimized. Prior work [28] has examined the influence of lighting, camera resolution, and background color on smartphone-based body composition estimates, and future studies should extend the present findings by using randomized or counterbalanced method order and by evaluating CV_app_ performance under less controlled real-world conditions.

Another limitation of this study was the dichotomous classification of participants as either racial/ethnic minorities or non-minorities. This categorization was used because the sample sizes within individual racial and ethnic categories were too small to support stable subgroup-specific modeling. However, this binary grouping is a coarse social-category proxy and does not capture phenotypic characteristics that may be more directly relevant to computer vision performance, such as skin tone, lighting contrast, body shape, body size, or regional fat distribution. Because skin pigmentation can vary widely within these broad categories, future research should measure and analyze actual skin pigmentation rather than relying solely on racial or ethnic identification. Females and different ethnic groups can have distinct fat distributions [48,49]. For example, woman may have more subcutaneous fat in hips/thighs for females and visceral vs subcutaneous ratios can vary by ancestry [48,49]. A 2D silhouette-based algorithm might not capture these patterns equally for all body types. If the CV_app_’s model assumes a certain body shape, it could incorrectly estimate in bodies that deviate from that norm [26]. Future validation studies should therefore include larger and more varied cohorts, including broader ranges of BMI, age, sex, race/ethnicity, skin tone, clothing conditions, and body morphology. Algorithmic fixes might involve multi-spectral imaging or depth sensing to reduce reliance on visible-light contrast [50]. Rigorous diversity and equity audits (e.g., testing on curated groups) should be standard practice during CV_app_ validation processes.

Future work should also evaluate mitigation strategies for reducing subgroup-dependent disagreement in CV-based body composition estimates. The most direct approach is to develop and validate models using larger, demographically and phenotypically diverse training datasets that include sufficient representation across sex, race/ethnicity, skin tone, body size, body shape, and body fat distribution. However, this approach requires access to the underlying algorithm and labeled training data, which are typically unavailable for proprietary commercial tools. A more immediately actionable strategy for independent researchers and practitioners is the development of post-hoc calibration models based on external validation datasets. For example, correction equations or calibration factors could be developed to adjust CV_app_-derived BF%, FM, or FFM estimates relative to a criterion or comparator method, either in the overall sample or within prespecified subgroups when sample size permits. From a model-development perspective, domain adaptation and fairness-aware training approaches may also help reduce performance gaps by explicitly accounting for differences in image-capture conditions, skin tone, body morphology, and subgroup-specific prediction error during model training and validation. Collectively, these approaches would help determine whether observed subgroup-dependent differences can be reduced through improved training data, external calibration, or algorithmic fairness methods before these tools are used for clinical decision-making or broad population screening.

## Conclusion

In summary, the current study highlights both the promise and limitations of smartphone computer vision-based body composition technology. The CV_app_ demonstrated excellent same-session repeatability under standardized laboratory conditions, indicating that it can produce highly consistent repeated values when image-capture conditions are controlled. However, the CV_app_ also showed systematic method-dependent differences compared with BODPOD and InBody, with patterns of disagreement that varied across sex and Minority analytic groups. Because the present study did not include a criterion reference method such as DXA or a 4-compartment model, these findings should be interpreted as evidence of relative disagreement among commonly used methods rather than definitive evidence of absolute measurement error. Even so, subgroup-dependent disagreement remains practically important because practitioners and consumers may interpret values from different body composition tools as interchangeable. The findings contrast with several prior studies [20,21,23,27,29] reporting strong agreement or minimal method-dependent differences for smartphone-based body composition tools, suggesting that not all CV_apps_ are equally robust across populations, comparator methods, or testing contexts. Importantly, these results reinforce that a digital measurement tool can be highly repeatable while still differing systematically from established comparator methods. Health professionals and researchers should therefore interpret CV_app_-derived estimates with caution, particularly when assessing diverse groups or making baseline, cross-sectional, clinical, or high-stakes decisions. Until additional criterion-validation studies and subgroup-specific evaluations are conducted, CV_app_-derived values should not be used as standalone diagnostic measures or treated as interchangeable with BODPOD, InBody, DXA, or 4-compartment model estimates. Future research should prioritize larger and more diverse validation samples, direct comparison with criterion methods, algorithm transparency, inclusive training datasets, and fairness-oriented evaluation to ensure equitable performance as digital anthropometry continues to evolve. These steps will be critical to ensure emerging digital health tools contribute positively to health equity rather than inadvertently widening disparities.

## Supporting information

S1 file Datafile. (XLSX)
