# Supplementary material for: Assessing body composition via a smartphone computer vision application: High repeatability but method-dependent agreement compared with BODPOD and Inbody
Source: PLOS Digit Health. 2026 Sep 2;5(9):e0000983. doi: 10.1371/journal.pdig.0000983 (PMC13537566; doi:10.1371/journal.pdig.0000983)
Supplement: Supplementary file 1 [file pdig.0000983.s002.pdf]

# Assessing Body Composition via a Smartphone Computer Vision Application: Reliable but Biased Compared to BODPOD and InBody

PDIG-D-25-00531_reviewer_JULIAN_BORGES

---

## Summary

This manuscript reports a repeated measures laboratory study comparing a smartphone based computer vision body composition application with air displacement plethysmography and bioelectrical impedance analysis. Outcomes include body fat percentage, fat mass, and fat free mass. The study demonstrates excellent test retest reliability across all methods and identifies systematic differences in estimates produced by the computer vision application, with larger discrepancies observed among female participants and among participants categorized as racial and ethnic minorities.

The topic is well aligned with the scope of PLOS Digital Health, addressing digital measurement validity, algorithmic accountability, and differential performance across demographic groups. The work is technically competent and transparently reported. However, several conceptual, statistical, and interpretive issues must be addressed to ensure that conclusions are appropriately bounded and framed within a rigorous measurement science context.

---

## Major Issues

### 1. Validation target and reference standard framing

The manuscript frequently implies accuracy or bias relative to an underlying ground truth while relying on BODPOD and InBody as comparator methods rather than criterion references. This creates ambiguity regarding the estimand and risks overstating conclusions about validity.

Recommendation

- Explicitly state in the Introduction and Methods that the study evaluates relative agreement across commonly used field methods under controlled conditions.
- Replace language implying absolute overestimation or underestimation with phrasing that reflects relative differences between methods.
- Add a concise explanation clarifying why systematic subgroup dependent disagreement remains important even without a criterion reference, while clearly bounding claims about accuracy.
- Distinguish clearly between repeatability and validity in the Discussion.

---

2. Fixed measurement order and potential conditioning effects

All participants completed assessments in the same fixed sequence. This introduces the possibility of conditioning effects, including posture familiarization, investigator guidance, and subtle physiologic or time dependent changes that may influence bioimpedance and image based measurements.

Recommendation

- Explicitly acknowledge fixed measurement order as a limitation and discuss its potential direction and magnitude of impact.
- If timing information is available, report elapsed time between measurements to contextualize the plausibility of physiologic drift.
- Temper comparative statements regarding relative reliability across methods, as determinism under repeated conditions is not equivalent to robustness.

---

3. Interpretation of near ceiling reliability estimates

The computer vision application demonstrates intraclass correlation coefficients at or near the ceiling. While high repeatability is expected under controlled conditions, such values may reflect deterministic algorithm output rather than true measurement stability across realistic sources of variation.

Recommendation

- State explicitly that high reliability reflects repeatability under tightly controlled capture conditions.
- Emphasize that reliability does not imply accuracy.
- Give greater interpretive weight to absolute reliability metrics such as standard error of measurement, coefficient of variation, and coefficient of reliability, and discuss their implications.

---

4. Multiplicity and analytic prioritization

The manuscript reports numerous outcomes, agreement metrics, and subgroup analyses. Although multiple comparison adjustments are applied in places, the inferential hierarchy is not clearly specified, increasing the risk of over interpretation of secondary findings.

Recommendation

- Define primary outcomes and primary contrasts a priori, with remaining analyses designated as exploratory.
- Establish and communicate a clear hierarchy of agreement metrics, prioritizing bias and limits of agreement and concordance correlation coefficient for agreement, and intraclass correlation coefficient with absolute error measures for reliability.
- Clarify the multiple testing strategy and the family of hypotheses being controlled.
- Consider relocating redundant metrics to supplementary material.

---

5. Race and ethnicity categorization and fairness interpretation

The binary categorization of participants as minority versus non minority is a pragmatic choice given sample size constraints but represents a coarse proxy for phenotypic features relevant to computer vision performance. Some discussion language implies causal attribution to race or ethnicity rather than differential performance across defined strata.

Recommendation

- Reframe findings as differential performance across the analytic strata used, without causal attribution to race or ethnicity.
- Strengthen the limitations by noting that skin tone, lighting contrast, and body shape distribution are plausible mediators that were not measured.
- Where feasible, provide descriptive subgroup detail while retaining the binary categorization for modeling.
- Use careful language when discussing equity implications, emphasizing potential risk rather than demonstrated downstream harm.

---

6. Clinical interpretation of observed differences

While the manuscript reports statistically significant differences and limits of agreement, the clinical relevance of these differences is insufficiently developed.

Recommendation

- Add a brief section translating observed differences in body fat percentage and fat mass into plausible clinical or fitness related decision consequences.
- Indicate whether the magnitude of disagreement could shift commonly used classification thresholds or screening decisions.
- Clearly state the use cases supported by the evidence, such as longitudinal within person tracking rather than baseline diagnostic assessment.

---

Minor Issues

1. Improve clarity by summarizing key capture conditions and device specifications in a concise table.
2. Ensure consistent terminology for body fat percentage, fat mass, and fat free mass throughout the manuscript.
3. Verify that interpretations of concordance correlation coefficient thresholds align with cited guidance.
4. Avoid conflating statistical significance with practical significance in the Discussion.
5. Clarify generalizability limits related to single site testing, controlled lighting, single phone model, and single application version.

---

Other Comments

With appropriate reframing, this manuscript has the potential to serve as a valuable example of rigorous evaluation of consumer facing digital measurement tools, particularly in demonstrating that high repeatability can coexist with systematic subgroup dependent disagreement.

---

Figures and Tables: Clearly presented and correctly labeled

Yes. Figures and tables are clear and appropriately labeled. Bland Altman plots effectively convey bias and limits of agreement, though they are information dense.

---

Methods: Sufficiently detailed?

Yes. The methods are described in sufficient detail to allow replication. Reporting of timing between measurements would further strengthen transparency.

---

Study Design: Appropriate for the question?

Partially. The design is appropriate for assessing repeatability and relative agreement under controlled conditions. It is not sufficient for absolute validity claims without a criterion reference.

---

Any experiments missing?

No additional experiments are required if claims are appropriately bounded. Inclusion of a criterion reference method would strengthen validity assessment and may be considered for future work.

---

Statistics: Statistical analyses sound?

Partially. The analyses are generally appropriate but require clearer prioritization of hypotheses, more transparent multiplicity handling, and more cautious interpretation of near ceiling reliability estimates.

---

Results and Data: Support conclusions?

The data support conclusions regarding high repeatability and systematic method dependent differences with subgroup variation. Claims regarding absolute bias and algorithmic fairness should be more carefully bounded.

---

All data accessible?

Yes. Data availability appears compliant with journal policy.

---

References: Anything missing?

The reference list is adequate. Additional references from the measurement science literature distinguishing repeatability from validity could further strengthen the Discussion.

---

Title: Appropriate and informative?

Yes. The title accurately reflects the core findings.
