# Supplementary material for: Assessing body composition via a smartphone computer vision application: High repeatability but method-dependent agreement compared with BODPOD and Inbody
Source: PLOS Digit Health. 2026 Sep 2;5(9):e0000983. doi: 10.1371/journal.pdig.0000983 (PMC13537566; doi:10.1371/journal.pdig.0000983)
Supplement: Supplementary file 2 [file pdig.0000983.s003.docx]

June 10, 2026

PLOS One Editor(s) and reviewers,

We are pleased to re-submit the following manuscript titled: “Assessing Body Composition via a Smartphone Computer Vision Application: High Repeatability but Method-Dependent Differences Compared with BODPOD and InBody”. With our resubmission we are providing a revised manuscript with changes noted in track changes and this response letter. Point by point responses to comments are provided below. We would like to express our appreciation to each of the reviewers and the editor for their time.

**Reviewer #1:** This is a well-executed and timely method comparison study. Please revise to clarify the validation target and frame findings as relative agreement rather than absolute accuracy, explicitly address fixed measurement order and its implications, interpret near ceiling reliability as repeatability under controlled conditions, prioritize primary outcomes and contrasts with clear multiplicity handling, refine demographic subgroup language, and translate observed differences into clinically meaningful context.

Major Issues

1. Validation target and reference standard framing

The manuscript frequently implies accuracy or bias relative to an underlying ground truth while relying on BODPOD and InBody as comparator methods rather than criterion references. This creates ambiguity regarding the estimand and risks overstating conclusions about validity.

Recommendation

- Explicitly state in the Introduction and Methods that the study evaluates relative agreement across commonly used field methods under controlled conditions.
- Replace language implying absolute overestimation or underestimation with phrasing that reflects relative differences between methods.
- Add a concise explanation clarifying why systematic subgroup dependent disagreement remains important even without a criterion reference, while clearly bounding claims about accuracy.
- Distinguish clearly between repeatability and validity in the Discussion.

**Response: We thank the reviewer for this important comment and recommendations. We agree that the original manuscript needed clearer framing regarding the validation target and reference standard. Specifically, because BODPOD and InBody are comparator methods rather than criterion reference methods, the present study should be interpreted as an evaluation of relative agreement and same-session repeatability rather than absolute accuracy or validity.**

**In response, we revised the Introduction, Methods, Results, Discussion, Practical Implications, Limitations, and Conclusion sections to clarify this framing. We now explicitly state that the study evaluated relative agreement across commonly used body composition methods under standardized laboratory conditions and was not designed to establish absolute accuracy against a criterion reference method such as DXA or a 4-compartment model.**

**We also revised wording throughout the manuscript to reduce language implying definitive overestimation, underestimation, or measurement error relative to ground truth. Instead, we now describe findings as method-dependent differences, relative disagreement, or higher/lower CVapp-derived values compared with BODPOD- or InBody-derived values.**

**We also added text explaining why subgroup-dependent disagreement remains important even without a criterion reference. Although this design cannot determine which method is closest to true body composition, method-dependent disagreement across sex or race/ethnicity analytic groups may still affect interpretation if practitioners or consumers treat values from different tools as interchangeable.**

2. Fixed measurement order and potential conditioning effects

All participants completed assessments in the same fixed sequence. This introduces the possibility of conditioning effects, including posture familiarization, investigator guidance, and subtle physiologic or time dependent changes that may influence bioimpedance and image based measurements.

Recommendation

- Explicitly acknowledge fixed measurement order as a limitation and discuss its potential direction and magnitude of impact.
- If timing information is available, report elapsed time between measurements to contextualize the plausibility of physiologic drift.
- Temper comparative statements regarding relative reliability across methods, as determinism under repeated conditions is not equivalent to robustness.

**Response: We thank the Reviewer for raising this important design consideration. Reviewer #3 had a similar comment. We agree that the fixed measurement order should be acknowledged more explicitly because it prevents potential order effects from being fully separated from method effects.**

**In response, we revised the Limitations section to state that all participants completed the assessments in the same fixed sequence: BODPOD, followed by InBody, followed by the CVapp.**

**This sequence was selected to standardize participant flow, operator procedures, and device setup within the single-session protocol. Because the assessments were passive and involved sitting quietly in the BODPOD, standing on the InBody platform, and standing for smartphone images, we considered meaningful fatigue-related carryover unlikely. However, we agree that a counterbalanced design would have allowed order effects to be evaluated directly. We therefore added language clarifying that method effects cannot be fully separated from potential order effects, and that the magnitude of any order-related influence cannot be quantified from the present data.**

**Additionally, timing between measurements was not recorded in a manner that would allow formal analysis of physiologic drift. Therefore, rather than adding a post-hoc estimate, we revised the manuscript to acknowledge this limitation directly. We also tempered language regarding reliability to clarify that the near-ceiling test–retest values reflect repeatability under tightly controlled same-session conditions, rather than robustness across varied testing conditions.**

3. Interpretation of near ceiling reliability estimates

The computer vision application demonstrates intraclass correlation coefficients at or near the ceiling. While high repeatability is expected under controlled conditions, such values may reflect deterministic algorithm output rather than true measurement stability across realistic sources of variation.

Recommendation

- State explicitly that high reliability reflects repeatability under tightly controlled capture conditions.
- Emphasize that reliability does not imply accuracy.
- Give greater interpretive weight to absolute reliability metrics such as standard error of measurement, coefficient of variation, and coefficient of reliability, and discuss their implications.

**Response: We thank the reviewer for this important recommendation. We agree that the near-ceiling ICC values should be interpreted carefully and should not be taken as evidence of accuracy, agreement with comparator methods, or robustness across varied testing conditions.**

**In response, we revised the Results, Discussion, and Practical Implications sections to clarify that the high ICC values reflect same-session repeatability under tightly controlled capture conditions.**

**Specifically, we added language stating that the ICC values indicate consistency between repeated measurements collected under standardized laboratory conditions, but that repeatability is distinct from accuracy or agreement with comparator methods.**

**We also revised the interpretation of the CVapp’s reliability to give greater attention to the absolute error metrics, including SEM, CoV, and CR. These metrics support that repeated CVapp measurements were highly consistent within the same session, while the agreement analyses showed that the repeated values were not necessarily interchangeable with BODPOD or InBody estimates.**

4. Multiplicity and analytic prioritization. The manuscript reports numerous outcomes, agreement metrics, and subgroup analyses. Although multiple comparison adjustments are applied in places, the inferential hierarchy is not clearly specified, increasing the risk of over interpretation of secondary findings.

Recommendation

- Define primary outcomes and primary contrasts a priori, with remaining analyses designated as exploratory.
- Establish and communicate a clear hierarchy of agreement metrics, prioritizing bias and limits of agreement and concordance correlation coefficient for agreement, and intraclass correlation coefficient with absolute error measures for reliability.
- Clarify the multiple testing strategy and the family of hypotheses being controlled.
- Consider relocating redundant metrics to supplementary material.

**Response: We thank the reviewer for this helpful recommendation. We agree that the analytic hierarchy and interpretation of multiple agreement metrics should be clearer to reduce the risk of overinterpreting secondary findings.**

**Because the overall analytic approach was appropriate and multiple comparison adjustments were already applied to pairwise method comparisons, we made targeted revisions to the Statistical Analysis section rather than restructuring the Results or relocating metrics to supplementary material.**

**Specifically, we clarified that BF%, FM, and FFM were the primary outcomes and that the primary method contrast was CVapp versus BODPOD, with CVapp versus InBody and BODPOD versus InBody treated as secondary contrasts.**

**We also clarified the hierarchy of agreement metrics, noting that Bland–Altman mean differences and limits of agreement, along with Lin’s CCC, were prioritized for agreement interpretation, whereas Pearson correlations, MAE, RMSE, and ICC values were considered complementary.**

**For reliability, we clarified that ICC values were interpreted alongside absolute error metrics, including SEM, CoV, and CR.**

**Finally, we specified that Bonferroni adjustments were applied within each outcome-specific family of pairwise method comparisons and that subgroup analyses were interpreted as secondary and exploratory.**

5. Race and ethnicity categorization and fairness interpretation

The binary categorization of participants as minority versus non-minority is a pragmatic choice given sample size constraints but represents a coarse proxy for phenotypic features relevant to computer vision performance. Some discussion language implies causal attribution to race or ethnicity rather than differential performance across defined strata.

Recommendation

- Reframe findings as differential performance across the analytic strata used, without causal attribution to race or ethnicity.
- Strengthen the limitations by noting that skin tone, lighting contrast, and body shape distribution are plausible mediators that were not measured.
- Where feasible, provide descriptive subgroup detail while retaining the binary categorization for modeling.
- Use careful language when discussing equity implications, emphasizing potential risk rather than demonstrated downstream harm.

**Response: We thank the reviewer for this important comment. We agree that the binary categorization of participants as racial/ethnic minority versus non-minority is a pragmatic but coarse analytic approach. We also agree that race and ethnicity should not be interpreted as causal mechanisms for differences in CVapp performance, particularly because phenotypic features more directly relevant to computer vision performance, such as skin tone, lighting contrast, body morphology, and regional fat distribution, were not directly measured.**

**In response, we revised the Participants, Statistical Analysis, Results, Discussion, Practical Implications, and Limitations sections to use more cautious language. Specifically, we now frame these findings as differences in method agreement across the analytic strata used in the present study, rather than as evidence that race or ethnicity directly caused differences in CVapp performance.**

**We also expanded the Participants section to provide descriptive subgroup detail while retaining the binary grouping for inferential modeling because several individual racial and ethnic categories had small cell sizes.**

**Finally, we strengthened the Limitations section to note that skin tone, lighting contrast, body shape, body size, and regional fat distribution are plausible contributors to computer vision performance but were not measured in this study.**

6. Clinical interpretation of observed differences

While the manuscript reports statistically significant differences and limits of agreement, the clinical relevance of these differences is insufficiently developed.

Recommendation:

- Add a brief section translating observed differences in body fat percentage and fat mass into plausible clinical or fitness related decision consequences.
- Indicate whether the magnitude of disagreement could shift commonly used classification thresholds or screening decisions.
- Clearly state the use cases supported by the evidence, such as longitudinal within person tracking rather than baseline diagnostic assessment.

**Response: We thank the reviewer for this helpful recommendation. We agree that the clinical and practical relevance of the observed method-dependent differences should be more clearly described. In response, we expanded the Practical Implications section to translate the observed differences in BF% and FM into plausible clinical, fitness, and occupational decision-making contexts.**

**Specifically, we now distinguish between lower-stakes screening or self-monitoring applications and diagnostic or high-stakes decision-making. We clarified that the CVapp may be useful for identifying individuals who may benefit from follow-up assessment or for tracking within-person trends over time, provided repeated measurements are collected under similar conditions and interpreted as approximate estimates rather than diagnostic values. However, we also note that observed differences, including CVapp-derived BF% values that were 3.9% higher than BODPOD in females and 2.7% higher than BODPOD among racial and ethnic minority participants, may be large enough to influence classification, counseling, or referral decisions when individuals are near clinical, fitness, or occupational thresholds. Therefore, we revised the manuscript to state that CVapp-derived values should not be used as standalone diagnostic measures or as the sole basis for obesity classification, nutrition counseling, intervention evaluation, or occupational fitness decisions without confirmation from a validated criterion or comparator method.**

1. Improve clarity by summarizing key capture conditions and device specifications in a concise table.

**Response: We thank the reviewer for this suggestion. We agree that summarizing the key device specifications and capture conditions improves clarity and helps readers interpret the standardized testing environment. In response, we added a concise table to the Methods section summarizing the key features of each measurement method, including device/application, manufacturer, measurement type, participant positioning, capture conditions, and primary outputs.**

2. Ensure consistent terminology for body fat percentage, fat mass, and fat free mass throughout the manuscript.

**Response: We thank the reviewer for noting this issue. We reviewed the manuscript for consistency in terminology related to body composition outcomes. In response, we standardized terminology throughout the manuscript so that the three primary outcomes are consistently referred to as body fat percentage (BF%), fat mass (FM), and fat-free mass (FFM). We also revised table notes, figure captions, and abbreviation lists to use consistent capitalization and hyphenation, particularly for fat-free mass. Where abbreviations are used, we now consistently use BF%, FM, and FFM after the terms are first defined. Also, we will note that for section headings the first letter in each term is capitalized.**

3. Verify that interpretations of concordance correlation coefficient thresholds align with cited guidance.

**Response: We thank the reviewer for noting this issue. We reviewed the concordance correlation coefficient interpretation thresholds and revised the manuscript to align the category labels with the cited guidance. Specifically, we replaced the prior wording of “>0.95 = excellent agreement” with the more specific categories of 0.95–0.99 = substantial agreement and >0.99 = almost perfect agreement. This change was made in both the Statistical Analysis section and the Figure 2 note to ensure consistency throughout the manuscript.**

4. Avoid conflating statistical significance with practical significance in the Discussion.

**Response: We agree that the Discussion should clearly distinguish statistical significance from practical or clinical significance.**

**In response, we reviewed the Results and Discussion sections and revised language where needed to avoid interpreting statistically significant findings as inherently practically meaningful.**

**Specifically, we added language noting that several statistically significant method effects were accompanied by small generalized eta-squared values and should therefore be interpreted alongside mean differences, pairwise effect sizes, agreement metrics, and limits of agreement.**

**We also expanded the Practical Implications section to discuss when the observed magnitude of method-dependent differences may be clinically relevant, particularly when individuals are near classification, counseling, or occupational decision thresholds.**

5. Clarify generalizability limits related to single site testing, controlled lighting, single phone model, and single application version.

**Response: We agree that the generalizability limits of the study should be clearly stated. We have addressed this in the revised Limitations section by clarifying that the CVapp was evaluated in a single laboratory setting under standardized imaging conditions using a fixed smartphone model, fixed tripod distance and height, plain white background, and consistent ambient lighting. Therefore, the findings should not be interpreted as representing performance across all possible home, field, device, or software-use conditions.**

**We also note that this standardized design was intentional because the goal of the present study was to evaluate relative agreement under controlled conditions while minimizing image-capture variability. However, we agree that future studies should examine performance across multiple sites, smartphone models, app versions, lighting conditions, backgrounds, clothing conditions, and home or field environments.**

With appropriate reframing, this manuscript has the potential to serve as a valuable example of rigorous evaluation of consumer facing digital measurement tools, particularly in demonstrating that high repeatability can coexist with systematic subgroup dependent disagreement.

**Response: We thank the reviewer for this thoughtful summary and constructive guidance. We agree that the manuscript is strongest when framed as an evaluation of relative agreement and same-session repeatability, rather than absolute accuracy.**

**In response to the comments of several reviewers, we revised the manuscript throughout to emphasize that high repeatability can coexist with systematic method-dependent and subgroup-dependent disagreement. We believe this reframing more accurately reflects the study design, strengthens the interpretation of the findings, and better highlights the importance of rigorous independent evaluation of consumer-facing digital measurement tools.**

**Reviewer #2:** The manuscript addresses a problem in digital health, namely the feasibility of smartphone-based computer vision (CV) for body composition estimation. The study is well-structured, with a clearly defined objective, appropriate statistical framework, and meaningful subgroup analyses. The use of multiple agreement metrics and reliability measures strengthens methodological rigor. Additionally, the focus on demographic bias (sex and minority groups) is a notable strength, aligning with current concerns in AI fairness and healthcare equity.

However, several critical limitations reduce the manuscript’s broader scientific impact.

First, the novelty is somewhat limited, as prior studies have already demonstrated both high reliability and demographic bias in CV-based body composition tools. The manuscript confirms existing findings rather than advancing methodological or algorithmic innovation. A deeper technical discussion of the CVapp model (e.g., architecture, training data distribution, feature extraction pipeline) is missing, which limits reproducibility and interpretability. Given that the observed bias is attributed to training data and algorithm design, the absence of such details weakens causal claims.

**Response: We thank the reviewer for this thoughtful comment. We agree that prior studies have demonstrated that smartphone- or image-based body composition tools can show high reliability and that performance may differ across demographic subgroups.**

**However, we respectfully note that the present study makes a distinct applied contribution by evaluating the relative agreement and same-session repeatability of a specific commercially available CVapp against two commonly used body composition methods in research, clinical, fitness, and applied settings: BODPOD and InBody. While previous studies have commonly compared CV-based methods with DXA or 4-compartment models, fewer data exist on whether these tools produce values that are comparable to methods that practitioners more commonly encounter in applied body composition assessment settings. Thus, the purpose of the present study was not to advance a new algorithm, but to provide an independent end-user validation of a commercially available tool under standardized conditions.**

**We also agree that additional technical details regarding the CVapp model architecture, training dataset distribution, preprocessing pipeline, segmentation approach, feature extraction strategy, and inference mechanisms would improve reproducibility and interpretability. However, the CVapp evaluated in this study is a proprietary commercial application, and these model-level details were not publicly disclosed by the developer. Therefore, we are unable to provide a mechanistic description of the internal algorithm beyond the user-facing acquisition workflow and output variables available to independent users.**

**We revised the Methods and Limitations sections to make this limitation explicit and to clarify that the study should be interpreted as an independent validation of relative agreement and repeatability rather than a model-development or algorithmic mechanism study.**

**We also agree with the reviewer that the absence of algorithmic details limits causal interpretation. In response, we revised the Discussion and Limitations sections to avoid implying that the observed subgroup-dependent method differences were definitively caused by training data imbalance or algorithm design. Instead, we now describe these as plausible mechanisms that require further investigation. We also added text emphasizing the need for future transparent model-development studies, controlled validation designs, fairness-aware training, domain adaptation, and post-hoc calibration approaches to better isolate and mitigate the sources of subgroup-dependent disagreement.**

Second, the choice of reference standards (BODPOD and InBody) is a concern. While widely used, both methods have known inaccuracies. The lack of comparison with gold-standard techniques such as DXA or 4-compartment models restricts the validity of conclusions regarding “bias.” This limitation is acknowledged but not sufficiently mitigated. A stronger experimental design would include at least one gold-standard modality.
**Response: We thank the reviewer for raising this important point. We agree that DXA and 4-compartment models are stronger criterion reference methods for establishing absolute body composition validity, and the absence of such a criterion measure limits conclusions regarding absolute accuracy or measurement bias. In response, we revised the manuscript to make this limitation clearer and to avoid overstating conclusions about validity.**

**The purpose of the present study was not to establish criterion validity against a gold-standard body composition model, but rather to evaluate the relative agreement and same-session repeatability of a commercially available CVapp compared with two methods commonly used in research, clinical, fitness, and applied settings (BODPOD and InBody). This applied comparison remains important because practitioners and consumers may encounter these methods in real-world settings and may be interested in whether values from an accessible CVapp are comparable to those from established laboratory or field methods.**

**To address the Reviewer’s concern, we revised the Introduction and Methods to explicitly state that BODPOD and InBody were treated as comparator methods, not criterion references. We also revised the Results and Discussion to describe findings as method-dependent differences or relative disagreement rather than absolute error. Finally, we strengthened the Limitations section to state that future studies should compare the CVapp directly with DXA or 4-compartment models to determine criterion validity.**

Third, the sample size (n=49) is relatively small for subgroup analysis, particularly when stratified by sex and minority status. The dichotomization of race into “minority vs non-minority” is overly simplistic and may obscure meaningful variability in body composition and imaging response across populations. This reduces the robustness of claims regarding fairness and bias.
**Response: We agree that the sample size was modest for subgroup analyses and that dichotomizing race and ethnicity as “minority” versus “non-minority” is a coarse analytic approach that may obscure meaningful variability across populations. We have revised the manuscript to more clearly acknowledge these limitations and to temper the interpretation of fairness-related findings.**

**In response, we expanded the Participants section to provide descriptive detail on the racial and ethnic composition of the sample while explaining why the binary grouping was retained for inferential analyses. Specifically, several individual racial and ethnic categories had small cell sizes, making more granular subgroup modeling statistically inappropriate in the present sample. We also revised the Statistical Analysis, Results, Discussion, Practical Implications, and Limitations sections to describe these findings as differences in method agreement across the available race/ethnicity analytic groups, rather than as evidence that race or ethnicity directly caused differences in CVapp performance.**

**We further strengthened the Limitations section to emphasize that race and ethnicity are imperfect proxies for phenotypic characteristics that may be more directly relevant to computer vision performance, including skin tone, lighting contrast, body morphology, body size, and regional fat distribution. We also clarified that future studies should include larger and more diverse samples with direct measures of these phenotypic characteristics and sufficient sample sizes to support more granular and intersectional analyses.**

Fourth, while the paper discusses computer vision approaches, it lacks integration with broader quantitative imaging paradigms such as radiomics. Radiomics focuses on extracting high-dimensional quantitative features (texture, shape, intensity distributions) from images, which parallels the feature extraction stage in CV-based body composition estimation. The absence of this connection limits the theoretical depth of the work.

**Response: We sincerely thank the reviewer for this helpful suggestion. We agree that the original manuscript did not sufficiently connect smartphone-based CV body composition estimation with broader quantitative imaging paradigms, including radiomics. We also agree that radiomics provides a useful conceptual framework for understanding how image-derived information can be transformed into quantitative features for prediction, and how issues such as feature robustness, reproducibility, domain shift, validation bias, and clinical translation are relevant to image-based digital health tools.**

**In response, we revised the Introduction to explicitly link CV-based body composition estimation with radiomics and broader quantitative imaging. Specifically, we added a new paragraph after the newly added computer-vision/object-detection paragraph in the Introduction. This paragraph explains that radiomics commonly extracts high-dimensional quantitative features related to shape, texture, intensity distributions, and spatial patterns from medical images, and that smartphone-based CV body composition tools similarly transform image-derived information, such as body boundaries, contours, proportions, and shape descriptors, into quantitative estimates of BF%, FM, and FFM.**

**We also reviewed and incorporated the recommended reference, “Radiomics in Medical Imaging: Methods, Applications, and Challenges,” and added it to the reference list. The revised text uses this reference to strengthen the theoretical grounding of the manuscript and to emphasize that concerns commonly discussed in radiomics, including robustness, reproducibility, domain shift, and validation bias, are also relevant when evaluating proprietary CV-based body composition tools across demographic subgroups.**

**At the same time, we were careful to avoid implying that the specific CVapp evaluated in this study uses a radiomics pipeline. Because the CVapp’s internal model architecture and feature extraction strategy are proprietary and were not publicly disclosed, we frame radiomics as a related quantitative imaging paradigm rather than as a confirmed description of the app’s internal algorithm.**

Specific Recommendation (Important)

The authors should explicitly incorporate and discuss the following reference:
“Radiomics in Medical Imaging: Methods, Applications, and Challenges”
This inclusion is important for several reasons:
Radiomics provides a well-established framework for quantitative feature extraction, which directly relates to how CV models infer body composition from images.
It would strengthen the methodological grounding by connecting CV-based anthropometric estimation with medical imaging analytics.

The discussion on feature robustness, reproducibility, and bias in radiomics literature can enrich the interpretation of observed demographic biases in the CVapp.
It would position the work within a broader quantitative imaging and AI ecosystem, increasing its relevance for a medical AI audience.
**Response: We sincerely thank the reviewer for this specific recommendation. As described in our response to the preceding comment, we revised the Introduction to better integrate CV-based body composition estimation with broader quantitative imaging paradigms, including radiomics. In making this revision, we specifically reviewed and incorporated the recommended reference, “Radiomics in Medical Imaging: Methods, Applications, and Challenges.”**

**Specifically, we added a new paragraph in the Introduction, immediately after the newly added paragraph on computer vision, object detection, and visual representation learning, and before the paragraph summarizing prior smartphone-based body composition validation studies. In this paragraph, we explain that radiomics provides a well-established framework for extracting high-dimensional quantitative features from images, including features related to shape, texture, intensity distributions, and spatial patterns. We further clarify that this framework parallels smartphone-based CV body composition estimation, in which image-derived body boundaries, contours, proportions, and shape descriptors are transformed into estimates of BF%, FM, and FFM.**

**We also used this revision to strengthen the interpretation of the present findings by noting that challenges emphasized in radiomics, including feature robustness, reproducibility, domain shift, validation bias, and clinical translation, are also relevant to the evaluation of proprietary CV-based body composition tools across demographic subgroups. At the same time, we were careful not to imply that the specific CVapp evaluated in this study uses a radiomics pipeline, because its internal architecture and feature extraction strategy are proprietary and were not publicly disclosed.**

A short paragraph in the Introduction or Discussion linking CV-based body composition estimation to radiomics-based feature engineering would significantly improve the paper.

**Response: We sincerely thank the reviewer for this helpful suggestion. As noted in our responses above, we added a new paragraph in the Introduction linking CV-based body composition estimation to radiomics-based quantitative feature engineering. Specifically, this paragraph was added immediately after the newly added computer-vision/object-detection paragraph and before the paragraph summarizing prior smartphone-based body composition validation studies.**

**In the revised text, we explain that radiomics extracts high-dimensional quantitative features from images, including features related to shape, texture, intensity distributions, and spatial patterns, and that this feature-engineering paradigm parallels CV-based body composition estimation, where image-derived body boundaries, contours, proportions, and shape descriptors are transformed into estimates of BF%, FM, and FFM.**

**We also discuss how radiomics-related concerns, including feature robustness, reproducibility, domain shift, validation bias, and clinical translation, are relevant to evaluating proprietary CV-based body composition tools across demographic subgroups.**

Additional Suggestions
Provide technical details of the CV model (CNN type, training dataset diversity, preprocessing pipeline).

**Response:** **We sincerely thank the reviewer for this suggestion. We agree that details such as CNN architecture, training dataset diversity, preprocessing procedures, and feature extraction pipeline would improve technical interpretability if they were available. However, as noted in our response to Reviewer #3, Comment 1, the CVapp evaluated in this study is a commercially available proprietary tool, and the developer does not publicly disclose the internal model architecture, training dataset composition, preprocessing pipeline, feature extraction strategy, or inference mechanisms.**

**In response, we revised the Computer Vision Smartphone App subsection of the Methods to provide the technical and procedural details that were available to independent users, including the app name and developer, smartphone model, camera resolution, image views collected, tripod distance and height, background, lighting conditions, demographic inputs entered into the app, and output variables. We also added explicit text clarifying that unavailable model-level details were proprietary and not publicly disclosed.**

**We further revised the Limitations and Future Research Directions section to identify this lack of algorithmic transparency as a limitation of the study and as a broader challenge in evaluating commercial AI-enabled health tools. We therefore frame the present work as an independent end-user validation study rather than a model-development or mechanistic algorithm-evaluation study.**

Discuss potential mitigation strategies (e.g., domain adaptation, fairness-aware training, calibration models).

**Response: We thank the reviewer for this helpful suggestion. We agree that the Discussion should provide more concrete mitigation strategies rather than only describing potential sources of subgroup-dependent disagreement.**

**In response, we added a paragraph to the Limitations and Future Research Directions section describing several potential strategies for reducing subgroup-dependent disagreement in CV-based body composition estimates. Specifically, we now discuss three complementary approaches.**

**First, we note that the most direct strategy is to develop and validate models using larger and more diverse training datasets that include adequate representation across sex, race/ethnicity, skin tone, body size, body shape, and body fat distribution.**

**Second, because the CVapp evaluated in this study is proprietary and its model architecture and training data are not publicly available, we added post-hoc calibration models as a more immediately actionable strategy for independent researchers and practitioners. Such calibration equations could be developed from external validation datasets to adjust CVapp-derived BF%, FM, or FFM estimates relative to a criterion or comparator method.**

**Third, we added domain adaptation and fairness-aware training as model-development strategies that may help reduce performance gaps by accounting for differences in image-capture conditions, skin tone, body morphology, and subgroup-specific prediction error during training and validation.**

Expand on clinical implications, especially whether bias levels are acceptable for screening vs diagnostic use.

**Response: We agree that the clinical and practical implications of the observed method-dependent differences should be more clearly interpreted in relation to likely use cases. In response, we expanded the Practical Implications section to distinguish between lower-stakes screening or self-monitoring applications and diagnostic or high-stakes decision-making.**

Improve clarity in reporting effect sizes vs statistical significance (some interpretations appear inconsistent).

**Response: We reviewed the Results and Discussion sections to ensure that statistical significance, effect size magnitude, and practical interpretation were reported consistently. We identified several places where language could be clarified to avoid implying that statistical significance alone indicates practical importance.**

**In response, we revised the Results section to clarify that the statistically significant main effects of method were accompanied by small generalized eta-squared values, and that interpretation should also consider pairwise effect sizes, mean differences, agreement metrics, and limits of agreement. We also corrected the description of the CVapp versus InBody FFM comparison. In the prior version, this comparison was described as statistically significant despite an adjusted p-value of 0.052. We revised the text to state that this comparison did not reach statistical significance after adjustment.**

**Finally, we revised the Discussion to more clearly distinguish statistically significant method-dependent differences from practical or clinical relevance.**

Consider including a visual pipeline diagram for the CVapp workflow.

**Response: We appreciate this suggestion from the reviewer. We agree that a visual pipeline diagram can be useful when the algorithmic workflow is known, particularly for studies involving model development or transparent computer vision pipelines. However, the CVapp evaluated in the present study is a commercially available proprietary tool, and the internal algorithmic workflow, including image preprocessing, body segmentation, feature extraction, model architecture, training data, and inference mechanisms, was not publicly disclosed by the developer. For this reason, we elected not to include a visual pipeline diagram of the CVapp’s computational workflow, as doing so would require assumptions about proprietary steps that we cannot verify and could misrepresent how the application processes images.**

**Reviewer #3:** Paper presents smartphone-based computer vision (CV) applications for body composition assessment, comparing their performance against established techniques such as BODPOD and InBody. The study is well-motivated, particularly given the increasing demand for accessible, low-cost health monitoring tools. The experimental design is methodologically sound, employing repeated-measures ANOVA, Bland–Altman analysis, and multiple agreement metrics (MAE, RMSE, CCC, ICC), which together provide a comprehensive evaluation framework. The inclusion of subgroup analyses across sex and racial/ethnic groups is a notable strength, addressing an important gap related to algorithmic bias and fairness in AI-driven healthcare tools.

However, several critical issues limit the technical depth and broader impact of the work. First, while the manuscript repeatedly refers to “computer vision” and briefly mentions convolutional neural networks (CNNs), it lacks a rigorous description of the underlying algorithmic pipeline used by the CVapp. There is no discussion of model architecture, feature extraction strategy, training dataset characteristics, or inference mechanisms. This omission weakens the technical contribution, especially for a digital health journal where methodological transparency is essential. Without such detail, it is difficult to assess whether the observed biases stem from model design, training data imbalance, or deployment constraints.

**Response: We sincerely thank the reviewer for this important comment. We agree that transparency regarding model architecture, feature extraction, training data composition, preprocessing, and inference mechanisms is highly relevant for interpreting the performance of computer vision–based digital health tools. We also agree that, without such information, it is not possible to determine whether the observed subgroup-dependent differences arise from model architecture, training dataset imbalance, image preprocessing, body segmentation, deployment constraints, or other algorithmic design choices.**

**In the present study, the CV_app_ evaluated was a commercially available smartphone application. Its internal model architecture, feature extraction pipeline, training dataset characteristics, and inference mechanisms are proprietary and were not publicly disclosed by the developer. Therefore, we are unable to provide architecture-level or training-data-level details beyond the user-facing acquisition protocol and output variables available to independent users. We have revised the manuscript to make this limitation explicit. Importantly, we also revised the Discussion to avoid causal language implying that the observed method-dependent differences were definitively caused by training data imbalance or algorithm design. Instead, we now describe these as plausible mechanisms that require further investigation.**

**We believe this limitation also highlights the value of independent validation studies of commercial digital health tools. Many tools used in clinical, fitness, and consumer health settings rely on proprietary algorithms. As with other commercial body composition technologies, such as bioelectrical impedance devices, users and practitioners often do not have access to the internal model. Independent evaluation under standardized conditions is therefore essential for determining whether the outputs are repeatable, whether they agree with commonly used comparator methods, and whether performance differs across participant subgroups.**

**In response to this comment, we revised the manuscript in three ways:**

1. **We expanded the Computer Vision Smartphone App subsection to clarify the available information about the CVapp workflow and to distinguish the observable acquisition protocol from unavailable proprietary algorithmic details.**
2. **We added a limitation explicitly stating that the app’s model architecture, training data distribution, feature extraction strategy, and inference mechanisms were not publicly available.**
3. **We revised the Discussion and Limitations sections to use more cautious language regarding possible mechanisms and interpretation of subgroup-dependent method disagreement, replacing causal and absolute-accuracy language with appropriately qualified phrasing such as “may reflect,” “could contribute to,” and “cannot determine.”**

Second, the literature review on computer vision is relatively superficial and lacks connection to foundational and modern developments in object detection and visual representation learning. The manuscript would benefit significantly from situating its approach within the broader evolution of computer vision—from classical feature-based methods (e.g., edge detection, shape descriptors) to deep learning-based approaches (e.g., CNNs, region-based detectors, transformer-based vision models). In particular, the authors should be asked to include and discuss the paper “From classical techniques to convolution-based models: A review of object detection algorithms”, as it provides essential context on how modern CV systems extract spatial and semantic features from images. Incorporating this reference would strengthen the theoretical grounding and help readers understand how body composition estimation relates to general object detection and representation learning paradigms.

**Response: We sincerely thank the reviewer for this helpful suggestion. We agree that the original literature review would benefit from a clearer connection between smartphone-based body composition estimation and the broader development of computer vision, object detection, and visual representation learning. In response, we revised the Introduction to situate CV-based body composition estimation within the transition from classical handcrafted-feature approaches to modern deep learning–based visual representation methods. Specifically, we added a discussion of how classical methods relied on features such as edges, contours, shape descriptors, and texture patterns, whereas CNN-based approaches learn hierarchical spatial and semantic representations directly from image data.**

**We also reviewed and incorporated the recommended reference, “From classical techniques to convolution-based models: A review of object detection algorithms,” and cited it in the revised Introduction. This addition helps clarify how body composition estimation relates conceptually to broader computer vision pipelines, in which visual information is transformed into quantitative features or predictions. At the same time, we were careful not to overstate the technical details of the specific CVapp evaluated in this study, because its internal architecture, training data, and feature extraction pipeline are proprietary and were not publicly disclosed.**

**Specifically, we added a new paragraph to the Introduction immediately after the paragraph describing how CV-based body composition tools extract body dimensions, contours, volumes, and other image-derived features from photographs. This new paragraph links CV-based body composition estimation to the broader evolution of object detection and visual representation learning, from handcrafted image features to CNN-based learned representations. We also added the recommended review article to the reference list.**

**Accordingly, the revised text now provides broader theoretical grounding for the CVapp while preserving the manuscript’s scope as an independent validation study of a commercial tool rather than a model-development study.**

Third, although the statistical analysis is comprehensive, the interpretation of agreement metrics lacks sufficient critical depth. For example, while ICC values are reported as excellent (>0.99), the manuscript does not adequately emphasize that high reliability does not imply accuracy—especially given the poor CCC values and systematic biases observed. This distinction should be more explicitly articulated to avoid misleading conclusions about the practical utility of the CVapp.

**Response: We thank the reviewer for this important comment. We agree that the distinction between repeatability and agreement/accuracy needed to be stated more explicitly. The high ICC values observed in the present study indicate that the CVapp produced highly consistent repeated values within the same session under standardized imaging conditions. However, these reliability results do not indicate that the CVapp values were accurate or interchangeable with comparator-method values. As the Reviewer notes, the agreement analyses, including CCC values, Bland–Altman results, and subgroup comparisons, demonstrated method-dependent differences despite excellent same-session repeatability.**

**In response, we revised the Results, Discussion, and Practical Implications sections to clarify this distinction. Specifically, we now state that the near-ceiling ICC values should be interpreted as evidence of same-session repeatability under tightly controlled testing conditions, rather than evidence of accuracy or agreement with comparator methods. We also revised the Discussion to emphasize that the CVapp can be highly repeatable while still differing systematically from BODPOD and InBody estimates. Finally, we revised the Practical Implications section to clarify that the CVapp may be most appropriate for tracking within-person trends under similar measurement conditions, but that high repeatability does not support its use as a standalone diagnostic or classification tool.**

Fourth, the study design introduces potential sources of bias that are not fully addressed. The fixed measurement order (BODPOD → InBody → CVapp) may introduce order effects or fatigue-related variability. Additionally, the controlled imaging conditions (white background, fixed lighting, standardized distance) limit ecological validity, as real-world users are unlikely to replicate such conditions. The authors acknowledge some of these issues but do not quantify their potential impact.

**Response: We thank the reviewer for raising these important study design considerations. We agree that the fixed measurement order and controlled imaging conditions should be addressed more explicitly, and we have revised the Limitations section accordingly.**

**All participants completed the assessments in the same fixed sequence: BODPOD, followed by InBody, followed by the CVapp. This order was selected to standardize participant flow, operator procedures, and device setup during the single-session protocol. Because the assessments were passive and involved sitting quietly in the BODPOD, standing on the InBody platform, and standing for smartphone images, meaningful fatigue-related variability was considered unlikely. However, we agree that a counterbalanced design would have allowed order effects to be evaluated directly. Therefore, we added text acknowledging that method effects cannot be fully separated from potential order effects in the present design. We also clarified that the magnitude of any order-related influence cannot be quantified from these data because timing and counterbalanced order data were not collected.**

**We also agree that the standardized laboratory imaging conditions do not reflect the full range of conditions likely to occur during home or field use. However, these conditions were intentionally selected to reduce environmental sources of image variability and evaluate relative agreement under optimized capture conditions. In this context, the controlled design was intended to determine whether method-dependent differences were present even when image-capture variability was minimized. We have revised the manuscript to clarify that the present findings should not be interpreted as representing CVapp performance across all possible real-world conditions. Rather, the findings provide a conservative evaluation of relative agreement under standardized conditions, with future studies needed to evaluate performance under less controlled home or field environments.**

Fifth, the handling of demographic variables, particularly the binary grouping of racial/ethnic categories, is overly simplistic and may obscure meaningful variability. Given that the study highlights algorithmic bias, a more granular analysis (e.g., skin tone, body shape distribution, or intersectional factors) would provide stronger evidence and align better with current best practices in fairness-aware AI.

**Response: We thank the reviewer for this thoughtful comment. We agree that the binary grouping of racial/ethnic categories is limited and may obscure meaningful variability in characteristics that are more directly relevant to computer vision performance, including skin tone, lighting contrast, body morphology, body size, and regional fat distribution. However, with the present sample size, more granular racial and ethnic subgroup modeling was not statistically appropriate because several individual categories included small numbers of participants. Therefore, the binary grouping was retained as a pragmatic analytic strategy, but we revised the manuscript to make the limitations of this approach clearer.**

**In response, we expanded the Participants section to report the descriptive racial and ethnic composition of the sample while explaining why the binary grouping was used for inferential analyses. We also revised the Statistical Analysis, Results, and Discussion sections to describe the findings as differences in method agreement across race/ethnicity analytic groups rather than as causal effects of race or ethnicity. In addition, we strengthened the Limitations section to acknowledge that future work should directly measure phenotypic and intersectional factors such as skin tone, lighting contrast, body shape, body size, regional fat distribution, sex, and race/ethnicity in larger samples.**

Finally, while the discussion appropriately highlights bias and equity concerns, it remains largely descriptive rather than mechanistic. The authors speculate on causes such as training data imbalance and segmentation challenges but do not provide empirical validation or ablation-style analysis. Future work should include controlled experiments to isolate these factors.

**Response: We thank the reviewer for this important comment. We agree that identifying the mechanisms underlying subgroup-dependent disagreement is essential for improving CV-based body composition tools. However, the present study was designed as an independent end-user validation study of a commercially available proprietary application, rather than a model-development or mechanistic algorithm-evaluation study. Because the CVapp’s internal model architecture, training dataset composition, preprocessing procedures, segmentation approach, feature extraction strategy, and inference mechanisms were not publicly disclosed, we are unable to conduct ablation-style analyses or directly test whether the observed subgroup-dependent differences were caused by training data imbalance, segmentation error, body-shape assumptions, skin-tone-related image contrast, or other algorithmic factors.**

**We agree with the reviewer that such analyses are an important next step, but they would require access to the underlying model and/or a separate controlled experimental design in which factors such as lighting, background, skin tone, body morphology, clothing, and camera characteristics are systematically varied while holding other variables constant. These data were not collected in the current study and cannot be generated retrospectively from the existing dataset.**

**To address this concern, we revised the Discussion and Limitations sections to avoid causal language and to frame these explanations as plausible mechanisms rather than demonstrated causes. We also added text emphasizing the need for future controlled experiments, transparent model-development studies, and fairness-oriented validation designs to isolate these mechanisms.**
