# Supplementary material for: Assessing body composition via a smartphone computer vision application: High repeatability but method-dependent agreement compared with BODPOD and Inbody
Source: PLOS Digit Health. 2026 Sep 2;5(9):e0000983. doi: 10.1371/journal.pdig.0000983 (PMC13537566; doi:10.1371/journal.pdig.0000983)
Supplement: Peer review history

# Peer review: Decision Letter 0

**DOI:** 10.1371/journal.pdig.0000983.r001

**Authors:** Po-Chih Kuo

22 Apr 2026

Response to Reviewers Revised Manuscript with Track Changes Manuscript **Journal Requirements:**

1. Please update your online Competing Interests statement. If you have no competing interests to declare, please state: "The authors have declared that no competing interests exist."

2. Please provide separate main figure files in .tif or .eps format only and ensure that all files are under our size limit of 10MB.

For more information about how to convert your figure files please see our guidelines: https://journals.plos.org/digitalhealth/s/figures

3. Please provide an Author Summary. This should appear in your manuscript between the Abstract and the Introduction, and should be 150–200 words long. The aim should be to make your findings accessible to a wide audience that includes both scientists and non-scientists. Sample summaries can be found on our website under Submission Guidelines: https://journals.plos.org/digitalhealth/s/submission-guidelines#loc-parts-of-a-submission

4. Some material included in your submission may be copyrighted. According to PLOS’s copyright policy, authors who use figures or other material (e.g., graphics, clipart, maps) from another author or copyright holder must demonstrate or obtain permission to publish this material under the Creative Commons Attribution 4.0 International (CC BY 4.0) License used by PLOS journals. Please closely review the details of PLOS’s copyright requirements here: PLOS Licenses and Copyright. If you need to request permissions from a copyright holder, you may use PLOS's Copyright Content Permission form.

Please respond directly to this email or email the journal office and provide any known details concerning your material's license terms and permissions required for reuse, even if you have not yet obtained copyright permissions or are unsure of your material's copyright compatibility.

Potential Copyright Issues:

Figure 1: Please confirm whether you drew the images / clip-art within the figure panels by hand. If you did not draw the images, please provide (a) a link to the source of the images or icons and their license / terms of use; or (b) written permission from the copyright holder to publish the images or icons under our CC-BY 4.0 license. Alternatively, you may replace the images with open source alternatives. See these open source resources you may use to replace images / clip-art:

- https://commons.wikimedia.org

- https://openclipart.org/

5. If the reviewer comments include a recommendation to cite specific previously published works, please review and evaluate these publications to determine whether they are relevant and should be cited. There is no requirement to cite these works unless the editor has indicated otherwise.

**Additional Editor Comments (if provided):** **Reviewers' Comments:**

**Comments to the Author**

1. Does this manuscript meet PLOS Digital Health’s publication criteria?

Reviewer #1: Partly

Reviewer #2: Partly

Reviewer #3: Yes

**********

2. Has the statistical analysis been performed appropriately and rigorously?

Reviewer #1: Yes

Reviewer #2: N/A

Reviewer #3: Yes

**********

3. Have the authors made all data underlying the findings in their manuscript fully available (please refer to the Data Availability Statement at the start of the manuscript PDF file)?

The PLOS Data policy

Reviewer #1: Yes

Reviewer #2: Yes

Reviewer #3: Yes

**********

4. Is the manuscript presented in an intelligible fashion and written in standard English?

Reviewer #1: Yes

Reviewer #2: No

Reviewer #3: Yes

**********

Reviewer #1: This is a well executed and timely method comparison study. Please revise to clarify the validation target and frame findings as relative agreement rather than absolute accuracy, explicitly address fixed measurement order and its implications, interpret near ceiling reliability as repeatability under controlled conditions, prioritize primary outcomes and contrasts with clear multiplicity handling, refine demographic subgroup language, and translate observed differences into clinically meaningful context.

Reviewer #2: The manuscript addresses a problem in digital health, namely the feasibility of smartphone-based computer vision (CV) for body composition estimation. The study is well-structured, with a clearly defined objective, appropriate statistical framework, and meaningful subgroup analyses. The use of multiple agreement metrics and reliability measures strengthens methodological rigor. Additionally, the focus on demographic bias (sex and minority groups) is a notable strength, aligning with current concerns in AI fairness and healthcare equity.

However, several critical limitations reduce the manuscript’s broader scientific impact.

First, the novelty is somewhat limited, as prior studies have already demonstrated both high reliability and demographic bias in CV-based body composition tools. The manuscript confirms existing findings rather than advancing methodological or algorithmic innovation. A deeper technical discussion of the CVapp model (e.g., architecture, training data distribution, feature extraction pipeline) is missing, which limits reproducibility and interpretability. Given that the observed bias is attributed to training data and algorithm design, the absence of such details weakens causal claims.

Second, the choice of reference standards (BODPOD and InBody) is a concern. While widely used, both methods have known inaccuracies. The lack of comparison with gold-standard techniques such as DXA or 4-compartment models restricts the validity of conclusions regarding “bias.” This limitation is acknowledged but not sufficiently mitigated. A stronger experimental design would include at least one gold-standard modality.

Third, the sample size (n=49) is relatively small for subgroup analysis, particularly when stratified by sex and minority status. The dichotomization of race into “minority vs non-minority” is overly simplistic and may obscure meaningful variability in body composition and imaging response across populations. This reduces the robustness of claims regarding fairness and bias.

Fourth, while the paper discusses computer vision approaches, it lacks integration with broader quantitative imaging paradigms such as radiomics. Radiomics focuses on extracting high-dimensional quantitative features (texture, shape, intensity distributions) from images, which parallels the feature extraction stage in CV-based body composition estimation. The absence of this connection limits the theoretical depth of the work.

Specific Recommendation (Important)

The authors should explicitly incorporate and discuss the following reference:

“Radiomics in Medical Imaging: Methods, Applications, and Challenges”

This inclusion is important for several reasons:

Radiomics provides a well-established framework for quantitative feature extraction, which directly relates to how CV models infer body composition from images.

It would strengthen the methodological grounding by connecting CV-based anthropometric estimation with medical imaging analytics.

The discussion on feature robustness, reproducibility, and bias in radiomics literature can enrich the interpretation of observed demographic biases in the CVapp.

It would position the work within a broader quantitative imaging and AI ecosystem, increasing its relevance for a medical AI audience.

A short paragraph in the Introduction or Discussion linking CV-based body composition estimation to radiomics-based feature engineering would significantly improve the paper.

Additional Suggestions

Provide technical details of the CV model (CNN type, training dataset diversity, preprocessing pipeline).

Discuss potential mitigation strategies (e.g., domain adaptation, fairness-aware training, calibration models).

Expand on clinical implications, especially whether bias levels are acceptable for screening vs diagnostic use.

Improve clarity in reporting effect sizes vs statistical significance (some interpretations appear inconsistent).

Consider including a visual pipeline diagram for the CVapp workflow.

Reviewer #3: Paper presents smartphone-based computer vision (CV) applications for body composition assessment, comparing their performance against established techniques such as BODPOD and InBody. The study is well-motivated, particularly given the increasing demand for accessible, low-cost health monitoring tools. The experimental design is methodologically sound, employing repeated-measures ANOVA, Bland–Altman analysis, and multiple agreement metrics (MAE, RMSE, CCC, ICC), which together provide a comprehensive evaluation framework. The inclusion of subgroup analyses across sex and racial/ethnic groups is a notable strength, addressing an important gap related to algorithmic bias and fairness in AI-driven healthcare tools.

However, several critical issues limit the technical depth and broader impact of the work. First, while the manuscript repeatedly refers to “computer vision” and briefly mentions convolutional neural networks (CNNs), it lacks a rigorous description of the underlying algorithmic pipeline used by the CVapp. There is no discussion of model architecture, feature extraction strategy, training dataset characteristics, or inference mechanisms. This omission weakens the technical contribution, especially for a digital health journal where methodological transparency is essential. Without such detail, it is difficult to assess whether the observed biases stem from model design, training data imbalance, or deployment constraints.

Second, the literature review on computer vision is relatively superficial and lacks connection to foundational and modern developments in object detection and visual representation learning. The manuscript would benefit significantly from situating its approach within the broader evolution of computer vision—from classical feature-based methods (e.g., edge detection, shape descriptors) to deep learning-based approaches (e.g., CNNs, region-based detectors, transformer-based vision models). In particular, the authors should be asked to include and discuss the paper “From classical techniques to convolution-based models: A review of object detection algorithms”, as it provides essential context on how modern CV systems extract spatial and semantic features from images. Incorporating this reference would strengthen the theoretical grounding and help readers understand how body composition estimation relates to general object detection and representation learning paradigms.

Third, although the statistical analysis is comprehensive, the interpretation of agreement metrics lacks sufficient critical depth. For example, while ICC values are reported as excellent (>0.99), the manuscript does not adequately emphasize that high reliability does not imply accuracy—especially given the poor CCC values and systematic biases observed. This distinction should be more explicitly articulated to avoid misleading conclusions about the practical utility of the CVapp.

Fourth, the study design introduces potential sources of bias that are not fully addressed. The fixed measurement order (BODPOD → InBody → CVapp) may introduce order effects or fatigue-related variability. Additionally, the controlled imaging conditions (white background, fixed lighting, standardized distance) limit ecological validity, as real-world users are unlikely to replicate such conditions. The authors acknowledge some of these issues but do not quantify their potential impact.

Fifth, the handling of demographic variables, particularly the binary grouping of racial/ethnic categories, is overly simplistic and may obscure meaningful variability. Given that the study highlights algorithmic bias, a more granular analysis (e.g., skin tone, body shape distribution, or intersectional factors) would provide stronger evidence and align better with current best practices in fairness-aware AI.

Finally, while the discussion appropriately highlights bias and equity concerns, it remains largely descriptive rather than mechanistic. The authors speculate on causes such as training data imbalance and segmentation challenges but do not provide empirical validation or ablation-style analysis. Future work should include controlled experiments to isolate these factors.

**********

what does this mean?). If published, this will include your full peer review and any attached files.

**Do you want your identity to be public for this peer review?**  If you choose “no”, your identity will remain anonymous but your review may still be made public.

For information about this choice, including consent withdrawal, please see our Privacy Policy

Reviewer #1: No

Reviewer #2: No

Reviewer #3: No

**********

**Figure resubmission:**

**Reproducibility:**

To enhance the reproducibility of your results, we recommend that authors of applicable studies deposit laboratory protocols in protocols.io, where a protocol can be assigned its own identifier (DOI) such that it can be cited independently in the future. Additionally, PLOS ONE offers an option to publish peer-reviewed clinical study protocols. Read more information on sharing protocols at https://plos.org/protocols?utm_medium=editorial-email&utm_source=authorletters&utm_campaign=protocols

Attachment Submitted filename: PDIG-D-25-00531_reviewer_JULIAN_BORGES.pdf

# Author comment: Author response to Decision Letter 1

**DOI:** 10.1371/journal.pdig.0000983.r002

20 Jun 2026

Attachment Submitted filename: Response To Reviewers.docx

# Peer review: Decision Letter 1

**DOI:** 10.1371/journal.pdig.0000983.r003

**Authors:** Po-Chih Kuo

20 Jul 2026

Assessing Body Composition via a Smartphone Computer Vision Application: High Repeatability but Method-Dependent Agreement Compared with BODPOD and InBody

PDIG-D-25-00531R1

Dear Dr. Martin,

We are pleased to inform you that your manuscript 'Assessing Body Composition via a Smartphone Computer Vision Application: High Repeatability but Method-Dependent Agreement Compared with BODPOD and InBody' has been provisionally accepted for publication in PLOS Digital Health.

Before your manuscript can be formally accepted you will need to complete some formatting changes, which you will receive in a follow-up email from a member of our team.

Please note that your manuscript will not be scheduled for publication until you have made the required changes, so a swift response is appreciated.

IMPORTANT: The editorial review process is now complete. PLOS will only permit corrections to spelling, formatting or significant scientific errors from this point onwards. Requests for major changes, or any which affect the scientific understanding of your work, will cause delays to the publication date of your manuscript.

If your institution or institutions have a press office, please notify them about your upcoming paper to help maximize its impact. If they'll be preparing press materials, please inform our press team as soon as possible -- no later than 48 hours after receiving the formal acceptance. Your manuscript will remain under strict press embargo until 2 pm Eastern Time on the date of publication. For more information, please contact digitalhealth@plos.org.

Thank you again for supporting Open Access publishing; we are looking forward to publishing your work in PLOS Digital Health.

Best regards,

Po-Chih Kuo, Ph. D.

Section Editor

PLOS Digital Health

***********************************************************

**Additional Editor Comments (if provided):**

**Reviewer Comments (if any, and for reference):**

Reviewer's Responses to Questions

**Comments to the Author**

Reviewer #3: All comments have been addressed

**********

publication criteria?

Reviewer #3: Yes

**********

3. Has the statistical analysis been performed appropriately and rigorously?

Reviewer #3: N/A

**********

4. Have the authors made all data underlying the findings in their manuscript fully available (please refer to the Data Availability Statement at the start of the manuscript PDF file)?

The PLOS Data policy

Reviewer #3: Yes

**********

5. Is the manuscript presented in an intelligible fashion and written in standard English?

Reviewer #3: Yes

**********

Reviewer #3: Author has made all the suggested changes.

**********

what does this mean?). If published, this will include your full peer review and any attached files.

**Do you want your identity to be public for this peer review?**  If you choose “no”, your identity will remain anonymous but your review may still be made public.

For information about this choice, including consent withdrawal, please see our Privacy Policy

Reviewer #3: No

**********
